# Tissue-resident macrophages in the intestine are long lived and defined by Tim-4 and CD4 expression

**DOI:** 10.1084/jem.20180019

**Published:** 2018-06-04

**Authors:** Tovah N. Shaw, Stephanie A. Houston, Kelly Wemyss, Hayley M. Bridgeman, Thomas A. Barbera, Tamsin Zangerle-Murray, Patrick Strangward, Amanda J.L. Ridley, Ping Wang, Samira Tamoutounour, Judith E. Allen, Joanne E. Konkel, John R. Grainger

**Affiliations:** 1 Manchester Collaborative Centre for Inflammation Research, University of Manchester, Manchester, England, UK; 2 School of Biological Sciences, Faculty of Biology, Medicine and Health, Manchester Academic Health Science Centre, University of Manchester, Manchester, England, UK; 3 Mucosal Immunology Section, Laboratory of Parasitic Diseases, National Institute of Allergy and Infectious Diseases, National Institutes of Health, Bethesda, MD; 4 Wellcome Trust Centre for Cell-Matrix Research, University of Manchester, Manchester, England, UK

## Abstract

A defining feature of resident gut macrophages is their high replenishment rate from blood monocytes attributed to tonic commensal stimulation of this site. In contrast, almost all other tissues contain locally maintained macrophage populations, which coexist with monocyte-replenished cells at homeostasis. In this study, we identified three transcriptionally distinct mouse gut macrophage subsets that segregate based on expression of Tim-4 and CD4. Challenging current understanding, Tim-4^+^CD4^+^ gut macrophages were found to be locally maintained, while Tim-4^–^CD4^+^ macrophages had a slow turnover from blood monocytes; indeed, Tim-4^–^CD4^–^ macrophages were the only subset with the high monocyte-replenishment rate currently attributed to gut macrophages. Moreover, all macrophage subpopulations required live microbiota to sustain their numbers, not only those derived from blood monocytes. These findings oppose the prevailing paradigm that all macrophages in the adult mouse gut rapidly turn over from monocytes in a microbiome-dependent manner; instead, these findings supplant it with a model of ontogenetic diversity where locally maintained subsets coexist with rapidly replaced monocyte-derived populations.

## Abstract (precis)

Intestinal macrophages represent the last tissue macrophages thought to entirely adhere to van Furth's decades-old continuous monocyte replenishment model. In this study, Shaw et al. identify a population of intestinal macrophages that are long lived and maintained independently of monocyte replenishment over long periods of time.

## Graphical Abstract

**Figure:**
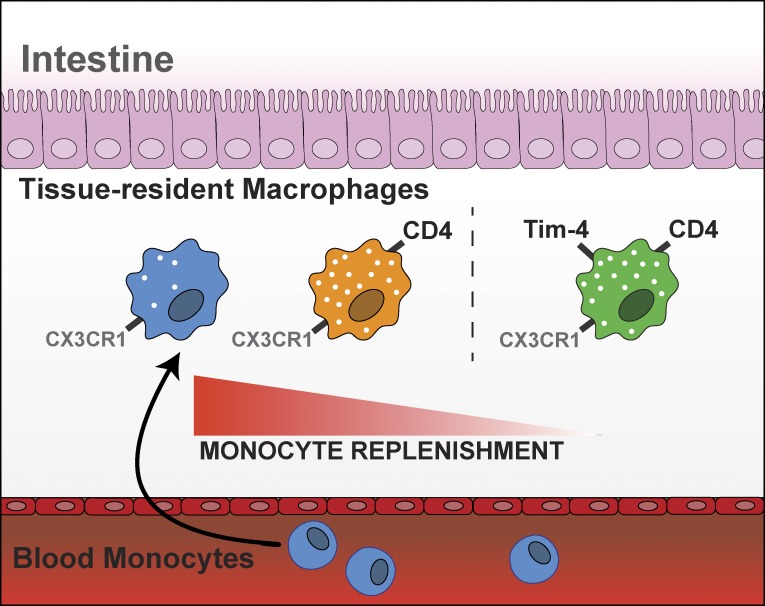

## Introduction

Resident gastrointestinal macrophages are a dominant immune cell type present in gut tissues that are crucial for homeostatic maintenance of this organ and hence its optimal physiological functioning (Bain and Mowat, 2014; Gross et al., 2015; Grainger et al., 2017). Key functions include clearance and sampling of apoptotic cells (Cummings et al., 2016; Schridde et al., 2017), instruction of epithelial progenitor proliferation in the intestinal crypts (Pull et al., 2005), bactericidal activity with limited inflammatory cytokine production (Smythies et al., 2005), and supporting neuroimmune interactions (Muller et al., 2014; Gabanyi et al., 2016). Inappropriate macrophage activity, particularly potential imbalance between resident- and inflammation-elicited (inflammatory) macrophages, has been implicated in driving pathophysiological complications in the gut. These include inflammatory bowel diseases (Kamada et al., 2008; Bain et al., 2013) and colon cancer (Afik et al., 2016). Delineating the origins and processes that underlie development of resident gut macrophages is therefore of high importance to provide novel mechanistic understanding of disease states.

For almost half a century, it was commonly believed that tissue-resident macrophages in all bodily organs were continuously renewed from adult bone marrow (BM)-derived circulating blood monocytes (van Furth et al., 1972). More recent research challenged this paradigm, revealing the presence of bona fide tissue-resident macrophages often arising from embryonic or perinatal precursors that are maintained locally and independently of blood monocytes at homeostasis (Ginhoux et al., 2010; Hoeffel et al., 2012, 2015; Schulz et al., 2012; Hashimoto et al., 2013; Yona et al., 2013; Sheng et al., 2015; Ginhoux and Guilliams, 2016). These include the brain microglia of the central nervous system (Ginhoux et al., 2010), alveolar macrophages of the lung airspaces (Guilliams et al., 2013; Hashimoto et al., 2013), and Langerhans cells of the skin epidermis (Merad et al., 2002; Hoeffel et al., 2012). Many of these organs are also home to blood monocyte–replenished macrophage populations that coexist with the locally maintained macrophages at homeostasis and can undertake functionally distinct activities (Ginhoux and Guilliams, 2016; Goldmann et al., 2016; Kim et al., 2016). The striking exception to this model has been the gastrointestinal tract, the last bastion of the continuous monocyte replenishment model of resident macrophage ontogeny, where all gut resident macrophages are replenished by high turnover from blood monocytes (Bain et al., 2014) with a predicted half-life of 4–6 wk (Jaensson et al., 2008; Ginhoux and Jung, 2014). This unique feature of resident gut macrophages has been attributed to the inflammatory tone of the gut stimulated by the high commensal burden (Bain et al., 2014; Ginhoux and Jung, 2014).

Unlike in other tissues, where multiple phenotypic markers in tandem with distinct morphological characteristics or localization (Merad et al., 2002; Guilliams et al., 2013; Tamoutounour et al., 2013; Bain et al., 2016; Scott et al., 2016) have been used to distinguish subsets of locally maintained resident macrophages from those that are monocyte replenished, gut studies have predominantly used relative expression of the chemokine receptor CX3C chemokine receptor 1 (CX3CR1; Jung et al., 2000; Varol et al., 2009; Tamoutounour et al., 2012; Zigmond et al., 2012; Bain et al., 2013, 2014). It therefore remains a distinct possibility that locally maintained resident macrophages or slowly monocyte-replenished populations could be present in the gut, but identification has been hampered by an inability to distinguish these cells from the total pool.

In this study, using the novel gut macrophage markers Tim-4 and CD4, we found that the adult gut macrophage pool is comprised of three similarly sized subsets with distinct replenishment rates from blood monocytes. Challenging current assumptions, abundant Tim-4^+^CD4^+^ gut-resident macrophages were found to be locally maintained independent of monocytes, whereas the Tim-4^–^CD4^+^ population had a slow replenishment rate from monocytes. Together, these two CD4^+^ populations accounted for the vast majority of mature macrophages in the gut. Indeed, the only population with high turnover from monocytes was the Tim-4^–^CD4^–^ macrophage subset. Supporting the differential requirement for monocyte replenishment, Tim-4^+^CD4^+^ macrophages had distinct developmental dynamics from early life and dominated in *Ccr2*^−/−^ animals, which have a paucity of circulating monocytes, whereas Tim-4^–^ subsets were dramatically depleted in these mice. Importantly, a live commensal microbiome is required for establishment of all resident macrophage subsets independent of ontogeny. These data redefine our understanding of gut macrophage development and heterogeneity at this critical mucosal site.

## Results and discussion

### Tim-4 and CD4 identify phenotypically and transcriptionally distinct populations of macrophages in the small intestine

Aiming to identify ontogenetically distinct macrophages within the total gut macrophage pool, we began by establishing candidate surface markers that have been used to distinguish mature or potentially long-lived macrophages in other studies and that are easily used for flow cytometry. We selected two such markers that are heterogeneously expressed on gut macrophages (Schridde et al., 2017) but have not been previously used to investigate ontogeny. One marker was the apoptotic cell-uptake receptor Tim-4, which is highly expressed by liver Kupffer cells (Scott et al., 2016) as well as the Gata-6–dependent large macrophages of the peritoneal cavity (Rosas et al., 2014; Bain et al., 2016; Kim et al., 2016). Both these macrophage populations dominate the compartment at birth and then have slow or little replenishment from blood monocytes in the adult (Bain et al., 2016; Scott et al., 2016). The other marker was CD4, recently identified as a gut-specific macrophage maturation marker based on transcriptional profiling of colonic versus skin macrophage development (Schridde et al., 2017).

**Figure 1.**
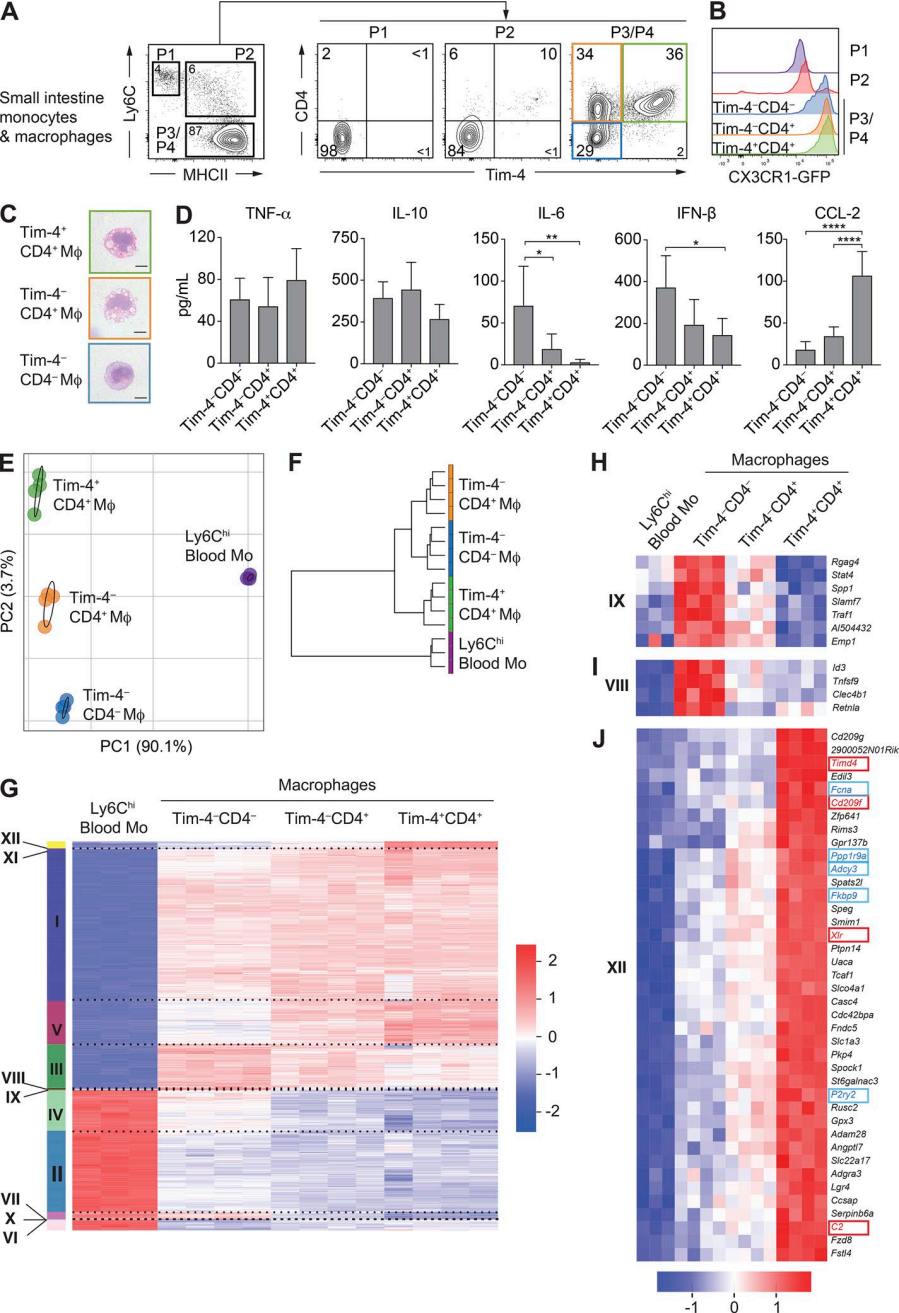
**Tim-4 and CD4 identify phenotypically and transcriptionally distinct populations of macrophages in the small intestine. (A)** Expression of Tim-4 and CD4 on small intestinal monocytes/macrophages assessed by flow cytometry from *Cx3cr1*^+/GFP^ reporter mice. Single-cell suspensions were first gated on live Lin^–^CD45^+^CD11b^+^CD11c^low/int^ cells, and then P1 monocytes (CD64^–^Ly6C^hi^MHCII^–^), P2 transitioning monocytes (Ly6C^+^MHCII^+^), and P3/P4 macrophages (CD64^+^Ly6C^–^MHCII^+^) were identified. Numbers denote the percentages of cells within the gate. Data are representative of at least three independent experiments. *n* = 2–3 per experiment. **(B)** Expression of CX3CR1-GFP by P1 monocytes, P2 transitioning monocytes, and Tim-4^–^CD4^–^, Tim-4^–^CD4^+^, and Tim-4^+^CD4^+^ (P3/P4) macrophages from the small intestine of adult CX3CR1^+/GFP^ mice. Data are representative of at least three independent experiments. *n* = 2–3 per experiment. **(C)** Morphological characteristics as assessed by H&E staining of Tim-4^–^CD4^–^, Tim-4^–^CD4^+^, and Tim-4^+^CD4^+^ macrophages (Mφ) sorted by FACS from the small intestine of 8–10-wk-old C57BL/6 WT mice. Bars, 5 µm. Data are representative of two independent sorts from three pooled mice. **(D)** Concentrations of TNF-α, IL-10, IL-6, IFN-β, and CCL-2 in supernatants from 18-h cultures in M-CSF–containing media of Tim-4^–^CD4^–^, Tim-4^–^CD4^+^, and Tim-4^+^CD4^+^ macrophages sorted by FACS from the pooled small intestines of four to six pooled 8–10-wk-old C57BL/6 WT mice. Concentrations were determined from duplicate or triplicate wells of 30,000 sorted macrophages. Data are representative of five separate experiments. Error bars show means ± SD. Statistical comparisons between macrophage subsets are shown. Statistical comparisons were performed with one-way ANOVA with Bonferroni’s multiple comparison test: *, P ≤ 0.05; **, P ≤ 0.01; ****, P ≤ 0.0001. **(E)** PCA of global gene expression from Ly6C^hi^ blood monocytes (Blood Mo) and Tim-4^–^CD4^–^, Tim-4^–^CD4^+^, and Tim-4^+^CD4^+^ resident macrophages isolated by FACS from the small intestine of 8–10-wk-old C57BL/6 WT mice. **(F)** Hierarchical cluster analysis of Ly6C^hi^ blood monocytes and Tim-4^–^CD4^–^, Tim-4^–^CD4^+^, and Tim-4^+^CD4^+^ small intestine macrophage populations based on global gene expression. **(G)** Gene expression profile of the 2,283 genes differentially expressed (p-adjusted < 1e^–30^) in Ly6C^hi^ blood monocytes, and Tim-4^–^CD4^–^, Tim-4^–^CD4^+^, and Tim-4^+^CD4^+^ macrophages sorted from the small intestine with clusters identified by k-means. **(H)** Gene expression profile of the seven genes forming cluster IX up-regulated in Tim-4^–^CD4^–^ and Tim-4^–^CD4^+^ macrophages compared with Ly6C^hi^ blood monocytes and Tim-4^+^CD4^+^ macrophages. **(I)** Gene expression profile of the four genes forming cluster VIII up-regulated in Tim-4^–^CD4^–^ macrophages compared with Ly6C^hi^ blood monocytes and Tim-4^–^CD4^+^ and Tim-4^+^CD4^+^ macrophages. **(J)** Gene expression profile of the 40 genes up-regulated in Tim-4^+^CD4^+^ macrophages compared with Ly6C^hi^ blood monocytes and Tim-4^–^CD4^–^ and Tim-4^–^CD4^+^ macrophages. Genes highlighted in blue are those previously identified as genes associated with a tissuewide resident macrophage signature. Genes highlighted in red are those described as distinguishing embryonically derived Kupffer cells from BM-derived Kupffer cells. **(E–J)** RNA sequencing results were generated from four independent sorts from the small intestines of three pooled mice (macrophages) and three independent sorts from the peripheral blood of three to four pooled mice (monocytes). See also Fig. S1 and Tables S1 and S2.

We began by assessing expression of Tim-4 and CD4 on the monocyte and macrophage populations defined by the already well-established “P1–P4 waterfall” model of gut monocyte/macrophage differentiation (Figs. 1 A and S1 A; Tamoutounour et al., 2012; Zigmond et al., 2012; Bain et al., 2013, 2014). In this model, Ly6C^hi^CX3CR1^int^ blood monocytes enter into the gastrointestinal tissue (P1) and progressively down-regulate Ly6C expression while up-regulating surface MHCII (P2) as they become gut macrophages (P3/P4). These MHCII^hi^ macrophages can be further segregated based on CX3CR1-GFP reporter expression into a smaller CX3CR1^int^ (P3) population and larger CX3CR1^hi^ (P4) population, with the CX3CR1^hi^-expressing cells being the most mature resident gut macrophages (Tamoutounour et al., 2012; Zigmond et al., 2012; Bain et al., 2013, 2014). Tim-4– and CD4-positive cells were mainly present in this P3/P4 macrophage gate (Fig. 1 A). Tim-4 and CD4 staining revealed three distinct populations of macrophages at approximately equal frequencies that accounted for the entire P3/P4 gate: Tim-4^–^CD4^–^ (blue), Tim-4^–^CD4^+^ (orange), and Tim-4^+^CD4^+^ (green) macrophages. All of these macrophage subsets were predominantly CX3CR1^hi^, although a small population of CX3CR1^int^ cells was evident in the Tim-4^–^CD4^–^ population, suggesting that this population alone may include some less-mature macrophages (Fig. 1 B; Schridde et al., 2017). Therefore, the established CX3CR1^hi^ resident gut macrophage population can be split into three distinct subpopulations based on their expression of Tim-4 and CD4.

To begin to explore whether Tim-4 and CD4 were markers of gut macrophage heterogeneity, we isolated the three subsets using FACS for morphological and functional analyses (Fig. 1, C and D). All subsets were found to have macrophage-like morphology but with Tim-4^–^CD4^–^ being visually smaller than the two CD4^+^ subsets (Tim-4^+^ and Tim-4^–^), which both displayed prominent vacuoles typical of phagocytically active macrophages (Fig. 1 C). After overnight culture, differential capacity to produce cytokines and chemokines was observed between the subsets (Fig. 1 D). In particular, production of the cytokines IFN-β and IL-6 was enriched in the Tim-4^–^CD4^–^ subset, whereas the Tim-4^+^CD4^+^ subset produced more of the monocyte-recruiting chemokine CCL-2 (Fig. 1 D). Production of gut macrophage–associated cytokines IL-10 and TNF-α were unchanged between subsets.

Based on this suggested heterogeneity within the gut macrophage pool, we undertook bulk RNA sequencing of the three macrophage subsets alongside their circulating Ly6C^hi^ blood monocyte precursors. As expected, principal component analysis (PCA) revealed that all three macrophage subsets were extremely distinct from circulating blood monocytes, supportive of the differentiation of all populations into macrophages (Fig. 1 E). Moreover, as shown by PCA (Figs. 1 E and S1 B), each of the macrophage subsets possessed a unique transcriptional profile distinct from the other macrophage subsets. Unsupervised clustering of populations by global gene-expression profiles was performed to generate a cluster tree delineating relationships between macrophage populations as well as blood monocytes. Using this analysis, the two Tim-4^–^ macrophage subsets (CD4^+^ and CD4^–^) were suggested to be more closely related to each other than to the Tim4^+^CD4^+^ subset (Fig. 1 F).

Pairwise comparisons of gene expression levels between each population (p-adjusted < 1^e–30^) identified 2,283 differentially expressed genes, the vast majority being up- or down-regulated in macrophages compared with monocytes. Unsupervised hierarchical k-means clustering of the differentially expressed genes was also performed, generating 12 clusters of genes with distinct expression characteristics (Figs. 1 G and S1 C) and associated gene ontology (GO) terms (Table S1). Many of the clusters (clusters I–VII, X, and XI) confirmed established transcriptional pathways of macrophage differentiation from blood monocytes as well as providing further evidence, in addition to recent research (Schridde et al., 2017), that CD4 expression on macrophages is associated with the most mature macrophages (Fig. S1 C and Table S2). In particular, cluster V, in which genes were progressively up-regulated from blood monocytes to Tim-4^–^CD4^–^ macrophages to the CD4^+^ subsets (Fig. S1 C), included *Lrrc4c*, *Dtx3*, *H2-M2*, and *Ocstamp*, genes already described as forming part of a mature colonic macrophage signature.

Of crucial relevance to better understand the heterogeneity between the three subsets of novel macrophages were the small clusters of genes that were distinctly regulated between each subset. These included cluster IX, consisting of genes up-regulated in both Tim4^–^ (CD4^+^ and CD4^–^) macrophage populations that included genes associated with cytokine production and phagocytosis such as *Stat4*, *Slamf7*, and *Traf1* (Fig. 1 H), and cluster VIII, consisting of genes more highly expressed in Tim4^–^CD4^–^ macrophages that included the canonical alternative activation factor *Retnla* (encoding for Relm-α; Loke et al., 2002) as well as the TGFβ-responsive transcription repressor *Id3* (Fig. 1 I; Nakatsukasa et al., 2015).

Most importantly, supporting the existence of unappreciated ontogenetically distinct macrophage subsets in the mouse gut were the 40 genes forming cluster XII that were highly up-regulated by Tim4^+^CD4^+^ macrophages (Fig. 1 J). This cluster was enriched for genes defined as signature genes for tissue-resident macrophages aligned from multiple tissues and included *Fcna*, *P2ry2*, *Adcy3*, *Fkbp9*, and *Ppp1r9a* (Fig. 1 J, blue highlight; Gautier et al., 2012). This strongly supported the possibility that as in other tissues (Rosas et al., 2014; Bain et al., 2016; Scott et al., 2016), these Tim-4^+^ cells are long-lived resident macrophages. Additionally, contained within this cluster were 4 out of 10 core signature genes including *Timd4*, previously identified as distinguishing long-lived embryonically derived Kupffer cells from recently BM-derived Kupffer cells, namely *Xlr*, *Cd209f*, and *C2* (Fig. 1 J, red highlight; Scott et al., 2016).

Collectively, these analyses demonstrate that in the small intestine, distinct populations of macrophages can be identified based on expression of Tim-4 and CD4, with CD4^+^ cells implicated as more mature than CD4^–^. Notably, Tim-4^+^CD4^+^ macrophages were enriched for transcripts associated with resident or embryonically derived macrophages in other tissues and thus could be a previously unidentified resident gut macrophage population that can be maintained independently of adult blood monocytes.

### Tim-4^+^ macrophages dominate in the neonatal small intestine, whereas Tim-4^–^ subsets are dependent on subsequent CCR2-dependent monocyte recruitment

We hypothesized that if the Tim-4^+^CD4^+^ macrophage population contains embryonically derived or locally maintained cells, and the Tim-4^–^ macrophages turn over from adult blood monocytes, then Tim-4^+^ and Tim-4^–^ subsets would exhibit distinct developmental kinetics from birth into adult life.

**Figure 2.**
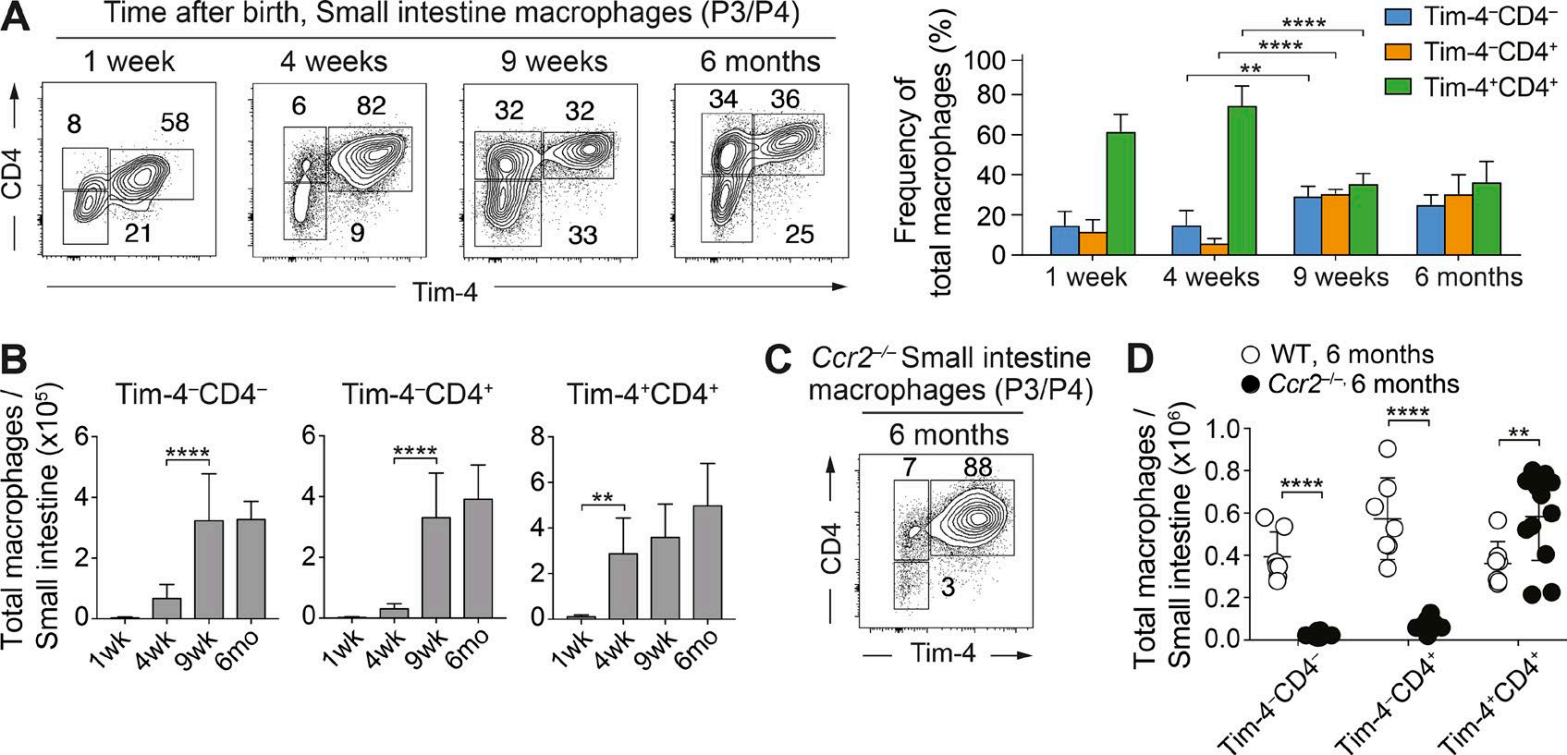
**Tim-4^+^CD4^+^ macrophages are present perinatally and are maintained in adulthood, whereas Tim-4^–^CD4^–^ and CD4^+^ macrophages are critically dependent on CCR2-mediated recruitment of monocytes. (A)** Representative flow cytometry plots (left) and frequencies (right) showing expression of Tim-4 and CD4 on live Lin^–^CD45^+^CD11b^+^CD11c^low/int^Ly6C^–^CD64^+^ P3/P4 macrophages in the small intestine of C57BL/6 WT mice at indicated ages. Data are representative of at least two independent experiments. *n* = 5–8 per group. Statistical comparisons between weeks 4 and 9 are shown. **(B)** Total number of small intestinal Tim-4^–^CD4^–^, Tim-4^–^CD4^+^, and Tim-4^+^CD4^+^ macrophages by age in C57BL/6 WT mice. Data are pooled from at least two independent experiments. *n* = 5–8 per group. Error bars show means ± SD. At 1 wk of age, intestines from two mice were pooled per sample. Statistical comparisons between weeks 1 and 4 and weeks 4 and 9 are shown. **(C)** Representative flow cytometry plots showing expression of Tim-4 and CD4 on P3/P4 macrophages in the small intestine of *Ccr2*^−/−^ mice at 6 mo of age. Data are representative of at least three independent experiments. Numbers in flow cytometry plots denote the percentages of cells within the gate. **(D)** Total number of Tim-4^–^CD4^–^, Tim-4^–^CD4^+^, and Tim-4^+^CD4^+^ macrophage subsets of *Ccr2*^−/−^ and C57BL/6 WT mice at 6 mo of age. Data are pooled from at least three independent experiments. *n* = 7–12 per group; results for individual animals are shown as dots. Statistical comparisons were performed with one-way ANOVA with Bonferroni’s multiple comparison test (A and B) or with two-way Student’s *t* test with Welch’s correction (D): **, P ≤ 0.01; ****, P ≤ 0.0001.

To this end, we analyzed the frequencies (Fig. 2 A) and absolute numbers (Fig. 2 B) of P3/P4 macrophages based on Tim-4 and CD4 expression from 1 wk after birth up to 6 mo old. Although total cell numbers were low relative to adulthood at the 1-wk time point (Fig. 2 B), Tim-4^+^CD4^+^ macrophages were the dominant cell population by frequency (Fig. 2 A), implicating a perinatal origin for at least some of this locally maintained population. This overall pattern was maintained at 4 wk of age, although by this time, Tim-4^+^CD4^+^ macrophages had expanded in absolute number in the tissue to adult numbers (Fig. 2 B). By 9 wk, all populations were approximately equally represented within the tissue, and this was associated with an increase in absolute numbers of both the Tim-4^–^CD4^+^ and CD4^–^ populations. Such temporal differences in expansion of the Tim-4^+^ (early) and Tim-4^–^ (late) subsets suggest different ontogenies, with Tim-4^+^ cells arriving early in life and Tim-4^–^ populations dependent on subsequent adult monocyte recruitment. The origin of the perinatal Tim-4^+^CD4^+^ macrophages could be fetal liver or early BM monocyte influx (<1 wk), but it is unlikely to be of yolk sac origin based on a previous study (Bain et al., 2014).

To probe these apparently distinct ontogenies further, we investigated Tim-4^+^ and Tim-4^–^ macrophage development in *Ccr2*^−/−^ animals (Boring et al., 1997). These animals have reduced circulating Ly6C^hi^ monocytes because of their defective exit from the BM, leading to a deficiency in monocyte-derived cell populations throughout the animal (Serbina and Pamer, 2006). In *Ccr2*^−/−^ animals, the Tim-4^+^CD4^+^ subset dominated the small intestinal macrophage compartment over the lifespan, whereas both Tim-4^–^ populations were sparsely represented (not depicted) even up to 6 mo of age when Tim4^–^ cells were abundant by frequency (Fig. 2 C) and cell number (Fig. 2 D). Thus, Tim-4^+^ and Tim-4^–^ macrophages have different requirements for CCR2, consistent with these two subsets having alternate ontogeny.

### Tim-4 and CD4 delineate macrophage populations with distinct dependence on blood precursors

**Figure 3.**
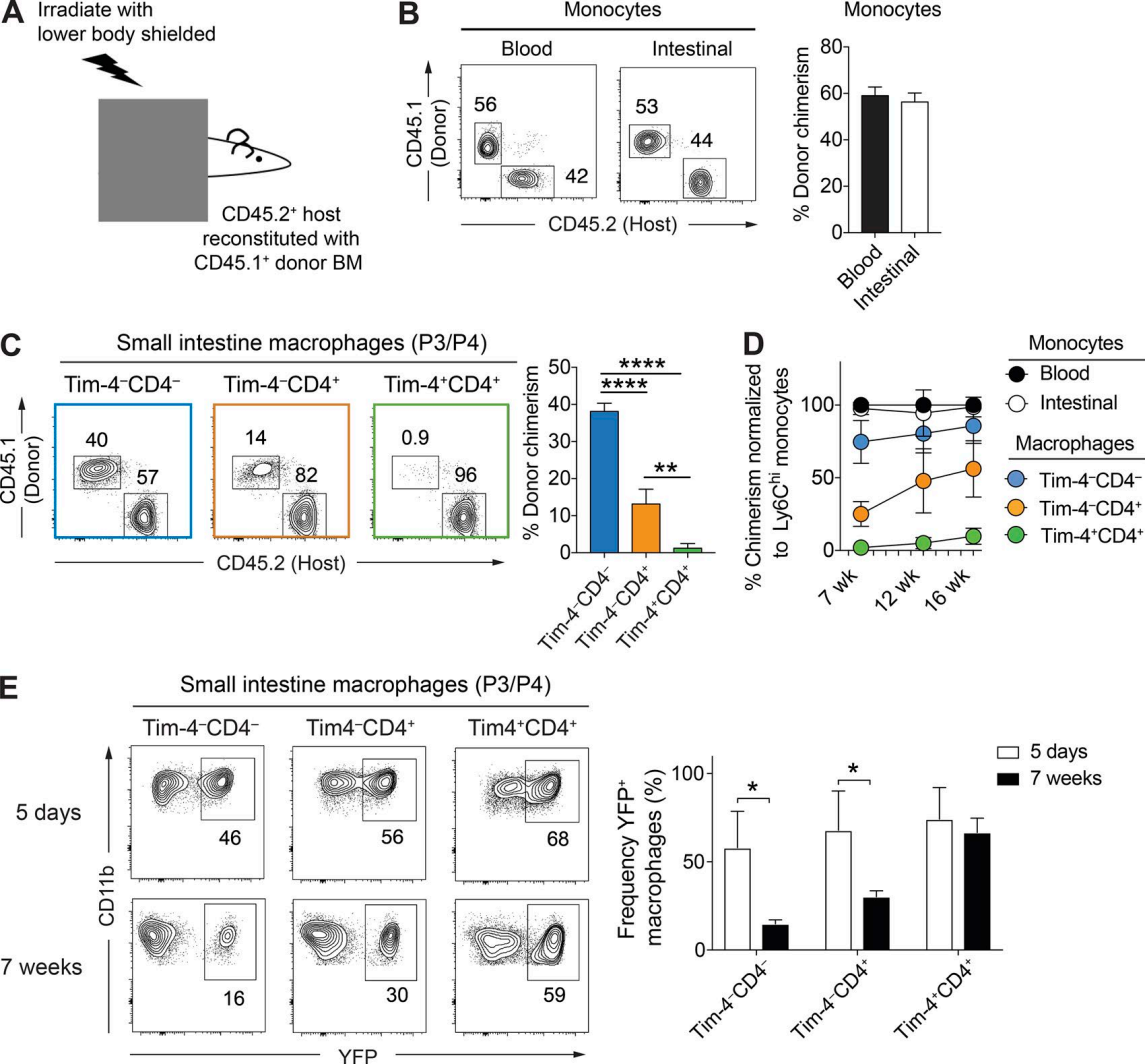
**Tim-4^+^CD4^+^ macrophages are infrequently replenished from blood monocytes, whereas Tim-4^–^ subsets are replenished at high and low rates. (A)** Schematic of gut-shielded chimera protocol to determine the contribution of blood monocytes to intestinal monocyte/macrophage subsets. C57BL/6 WT hosts aged 6–8 wk old were anaesthetized and positioned beneath a lead sheet shielding the lower two thirds of the body, including the intestine, from irradiation. Mice were reconstituted with donor BM cells from congenic CD45.1^+^ WT donor animals. **(B)** Left: Representative flow cytometry plots showing the frequency of CD45.1^+^ donor-derived cells within Ly6C^hi^ monocytes of the peripheral blood and small intestine of shielded chimeric mice 7 wk after irradiation. Right: Frequency of CD45.1^+^ donor-derived cells within the Ly6C^hi^ monocytes of the peripheral blood and small intestine of shielded chimeric mice 7 wk after irradiation. Data are representative of at least two independent experiments. *n* = 6 per group. **(C)** Left: Representative flow cytometry plots showing the frequency of CD45.1^+^ donor-derived cells within the Tim-4^–^CD4^–^, Tim-4^–^CD4^+^, and Tim-4^+^CD4^+^ macrophage subsets of the small intestine of shielded chimeric mice 7 wk after irradiation. Right: Frequency of CD45.1^+^ donor-derived cells within the Tim-4^–^CD4^–^, Tim-4^–^CD4^+^, and Tim-4^+^CD4^+^ macrophage subsets of the small intestine of shielded chimeric mice 7 wk after irradiation. Data are representative of at least two independent experiments. *n* = 6 per group. **(D)** At 7, 12, and 16 wk after irradiation, the frequency of donor-derived cells was determined in the intestinal Ly6C^hi^ monocyte and macrophage subpopulations of the small intestine by flow cytometry and normalized to the chimerism of Ly6C^hi^ blood monocytes. Data are pooled from at least two independent experiments above. *n* = 6 per group. **(E)** Left: Representative flow cytometry plots showing the frequency of YFP-expressing cells within the Tim-4^–^CD4^–^, Tim-4^–^CD4^+^, and Tim-4^+^CD4^+^ macrophage subsets of the small intestine of*Cx3cr1^CreER^* X *R26-yfp* mice 5 d and 7 wk after tamoxifen treatment. Right: Frequency of YFP-expressing cells within the Tim-4^–^CD4^–^, Tim-4^–^CD4^+^, and Tim-4^+^CD4^+^ macrophage subsets of the small intestine of *Cx3cr1^CreER^* X *R26-yfp* mice 5 d and 7 wk after tamoxifen treatment. Numbers in flow cytometry plots denote the percentages of cells within the gate. Data are pooled from two independent experiments. *n* = 4–5 per group. Error bars show means ± SD. Statistical comparisons were performed with one-way ANOVA with Bonferroni’s multiple comparison test (C) or with two-way Student’s *t* test with Welch’s correction (E): *, P ≤ 0.05; **, P ≤ 0.01; ****, P ≤ 0.0001. See also Fig. S2.

Our results thus far point to Tim-4^+^ and Tim-4^–^ macrophages having different requirements for adult monocyte replenishment. To more comprehensively explore the ontogenetic relationships of small intestinal macrophages, we used a gut-shielded BM chimera approach. BM chimeras were generated by a process in which WT CD45.2 mice were irradiated with their abdomen shielded from irradiation (Fig. 3 A). Animals were then reconstituted with congenic (CD45.1^+^) WT BM. Shield irradiation aimed to limit confounding effects of irradiating monocyte/macrophage populations in the gut and prevent development of a local inflammatory response. At 7 wk after irradiation, frequencies and numbers of all macrophage subsets in chimeric animals were similar to nonirradiated controls, indicating that shielding had prevented sustained perturbation of the tissue in these animals (Fig. S2 A).

As expected, chimerism of small intestinal Ly6C^hi^ monocytes was similar to blood Ly6C^hi^ monocytes by 7 wk after irradiation (Fig. 3 B). Strikingly, the degree of chimerism varied between the three novel macrophage populations (Fig. 3 C). Tim-4^–^CD4^–^ cells had the highest chimerism, implying that these cells were rapidly replenished from blood precursors. Contrasting this, Tim-4^–^CD4^+^ macrophages had much lower chimerism (<50% that of Tim-4^–^CD4^–^ macrophages), suggesting that this population was turned over much more slowly from blood monocytes. Of note, Tim-4^+^CD4^+^ macrophages had a chimerism of <1%, indicating that these macrophages were locally maintained.

To understand the temporal dynamics of this replenishment, we investigated the chimerism at later time points. Over time, the Tim-4^–^CD4^–^ and Tim-4^–^CD4^+^ macrophages continued to incorporate cells from the donor, although at different rates, with Tim-4^–^CD4^–^ cells having almost equivalent ratios of donor populations to blood monocytes as early as 12 wk (Fig. 3 D), whereas even at 16 wk, the Tim-4^–^CD4^+^ population had only achieved ∼50% of their potential chimerism (Fig. 3 D). Most importantly, in the Tim-4^+^CD4^+^ population, although donor cells were increased, they still constituted <5% of this macrophage subset in the small intestine (Fig. 3 D), further supporting their local maintenance and rare replenishment from blood monocytes.

As an alternative way of exploring the replenishment of resident gut macrophages, we generated reporter animals by crossing *Cx3cr1^CreER^* mice (Yona et al., 2013) to *R26-yfp* mice (Srinivas et al., 2001). In these animals, administration of tamoxifen allows translocation of the cre enzyme into the nucleus and irreversibly induces YFP expression by CX3CR1^+^ cells, strongly labeling CX3CR1^+^ gut macrophages (Yona et al., 2013). Adult animals 8–9 wk of age were given tamoxifen orally for five consecutive days to ensure robust and irreversible induction of YFP (Fig. S2 B). Animals were left for 5 d or 7 wk after the final tamoxifen dose to assess gut macrophage replenishment by blood monocytes. Any cells developing from blood monocytes after withdrawal of tamoxifen will express the cre enzyme latently in the cytoplasm such that they remain YFP^–^. Agreeing with published data (Yona et al., 2013), in this setting, Ly6C^low^ blood monocytes expressed YFP 5 d after the final tamoxifen dose before being replaced by YFP^–^ monocytes at 7 wk (Fig. S2, C and D), whereas CX3CR1^+^ microglia, which do not undergo replenishment by blood monocytes, maintained a high level of YFP expression at 7 wk (Fig. S2, C and D). In the gut of these animals correlating with data from the gut shield BM chimeras, replacement of Tim-4^–^CD4^–^ macrophages was higher than in Tim-4^–^CD4^+^ population, whereas there was extremely limited replacement of Tim-4^+^CD4^+^ cells from YFP^–^ monocytes (Fig. 3 E).

Together, using two independent strategies to investigate ontogeny, these results establish that phenotypically distinct populations of macrophages in the gut have dramatically different turnover from circulating monocytes in the adult animal. Either because of their longevity or self-renewal, Tim-4 marks a previously unappreciated locally maintained population of resident gut macrophages present in the small intestine.

### Locally maintained Tim-4^+^CD4^+^ macrophages are present in commensal-rich areas of the gut and are regulated by live microbiota

Although the concept of rapid continuous monocyte replenishment of resident macrophages has been applied to all gut macrophages, the vast majority of studies have focused on the colon, where this replenishment has been attributed to the high commensal burden (Bain et al., 2014; Ginhoux and Jung, 2014; Ginhoux and Guilliams, 2016). The small intestine, however, is relatively devoid of commensal flora caused in part by stomach acid, bile, and pancreatic secretions (O’Hara and Shanahan, 2006). After our identification of a long-lived Tim-4^+^CD4^+^ macrophage in the small intestine present from birth, we sought to understand whether development of this population was impaired in a commensal-rich region of the gut, where one would predict that monocyte-replenished populations would be favored.

**Figure 4.**
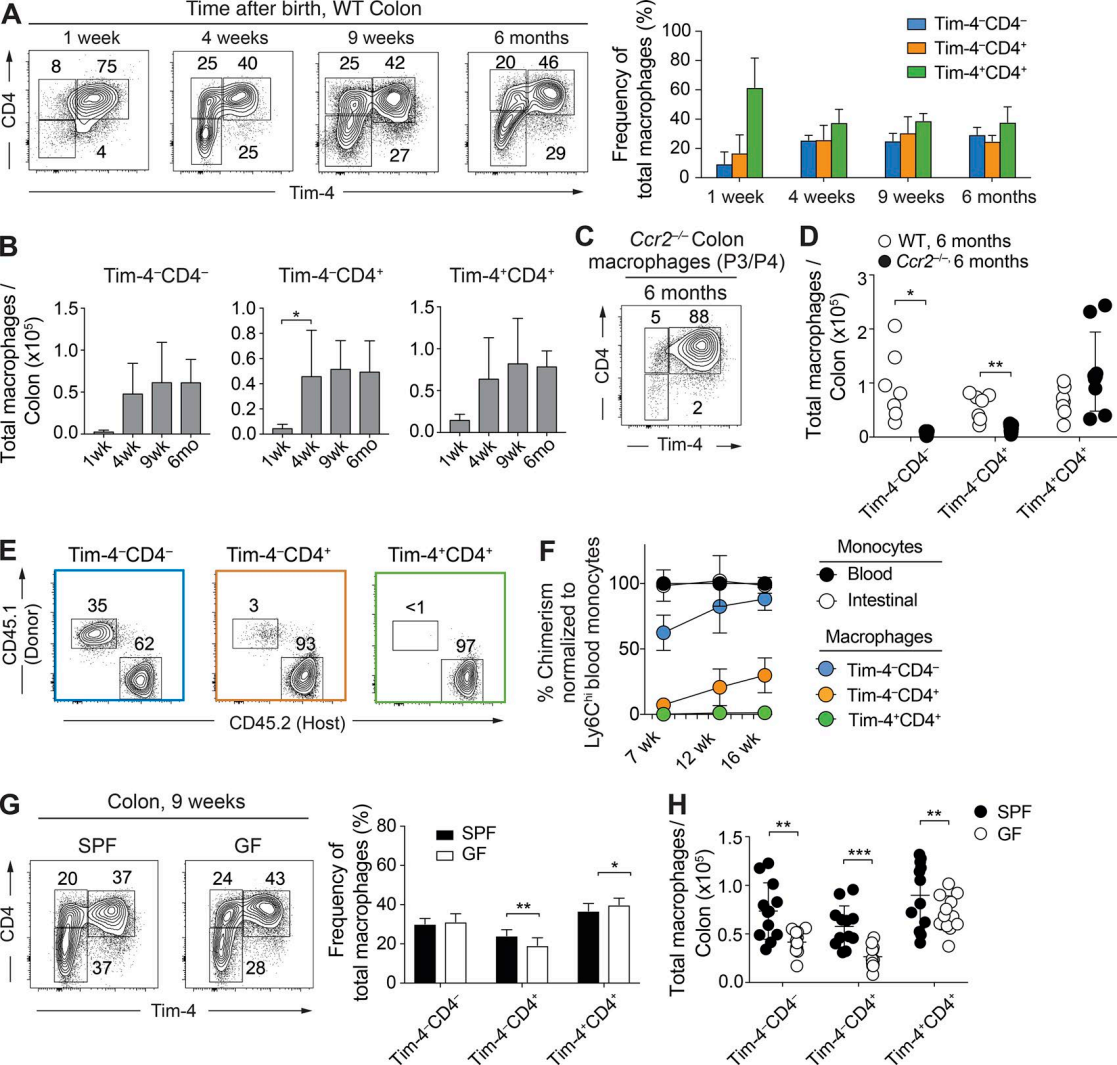
**Locally maintained Tim-4^+^CD4^+^ macrophages persist in commensal-rich areas of the gut and are regulated by a live microbiota. (A)** Representative flow cytometry plots (left) and frequency graph (right) showing expression of Tim-4 and CD4 on live Lin^–^CD45^+^CD11b^+^CD11c^low/int^Ly6C^–^CD64^+^ P3/P4 macrophages in the colon of C57BL/6 WT mice at the indicated ages. Data are representative of at least two independent experiments. *n* = 5–8 per group. **(B)** Total number of Tim-4^–^CD4^–^, Tim-4^–^CD4^+^, and Tim-4^+^CD4^+^ macrophage subsets by age in the colon of C57BL/6 WT mice. Data are pooled from at least two independent experiments. *n* = 5–8 per group. At 1 wk of age, intestines from two mice were pooled per sample. **(C)** Representative flow cytometry plots showing expression of Tim-4 and CD4 on P3/P4 macrophages in colon of *Ccr2*^−/−^ mice at 6 mo of age. Data are representative of at least three independent experiments. **(D)** Total number of Tim-4^–^CD4^–^, Tim-4^–^CD4^+^, and Tim-4^+^CD4^+^ macrophage subsets of *Ccr2*^−/−^ and C57BL/6 WT mice at 6 mo of age. Data are pooled from at least three independent experiments. *n* = 5–9 per group; results for individual animals are shown as dots. **(E)** Representative flow cytometric plots showing the frequency of CD45.1^+^ donor-derived cells within the Tim-4^–^CD4^–^, Tim-4^–^CD4^+^, and Tim-4^+^CD4^+^ macrophage subsets of the colon of shielded chimeric mice generated as described in Fig. 3 A 7 wk after irradiation (left). Frequency of CD45.1^+^ donor-derived cells within the Tim-4^–^CD4^–^, Tim-4^–^CD4^+^, and Tim-4^+^CD4^+^ macrophage subsets of the small colon of shielded chimeric mice 7 wk after irradiation (right). Data are representative of at least two independent experiments. *n* = 5 per group. **(F)** At 7, 12, and 16 wk after irradiation, the frequency of donor-derived cells was determined in the intestinal Ly6C^hi^ monocyte and macrophage subpopulations of the colon by flow cytometry and normalized to the chimerism of Ly6C^hi^ blood monocytes. Data generated as described in Fig. 3 A are pooled from at least two independent experiments. *n* = 5–6 per group. **(G)** Left: Representative flow cytometry plots showing expression of Tim-4 and CD4 on P3/P4 macrophages in the colon of SPF and GF WT C57BL/6 mice at 9 wk of age. Right: Frequency of Tim-4^–^CD4^–^, Tim-4^–^CD4^+^, and Tim-4^+^CD4^+^ macrophage subsets in the colon of 9-wk-old SPF and GF mice. Numbers in flow cytometry plots denote the percentages of cells within the gate. Data are pooled from four independent experiments. *n* = 12 per group. **(H)** Total number of Tim-4^–^CD4^–^, Tim-4^–^CD4^+^, and Tim-4^+^CD4^+^ macrophages in the colon of SPF and GF mice at 9 wk of age. Data are pooled from four independent experiments. *n* = 12 per group; results for individual animals are shown as dots. Error bars show means ± SD. Statistical comparisons were performed with one-way ANOVA with Bonferroni’s multiple comparison test (B) or with two-way Student’s *t* test with Welch’s correction (D, G, and H): *, P ≤ 0.05; **, P ≤ 0.01; ***, P ≤ 0.001. See also Fig. S3.

To this end, we began by investigating development of macrophages over early life into adulthood in the colon. Early after birth (1 wk), as observed in the small intestine (Fig. 2 A), Tim-4^+^CD4^+^ macrophages dominated (Fig. 4 A). At 4 wk after birth, unlike in the small intestine, Tim-4^–^ macrophage populations in the colon were already expanded to adult levels (Fig. 4, A and B). This correlated with the previously reported influx of monocyte-derived macrophages that replaced embryonically derived cells (Bain et al., 2014). Unexpectedly, however, the Tim-4^–^ monocyte-derived populations did not replace the Tim-4^+^CD4^+^ macrophages with time as Tim-4^+^CD4^+^ macrophages persisted even in 6-mo-old animals (Fig. 4, A and B), suggesting that even in the colon, the Tim-4^+^CD4^+^ macrophage population is dominant and long lived. As observed in the small intestine, macrophages with a Tim-4^–^ phenotype were critically dependent on expression of CCR2 as these populations were almost entirely absent in 6-mo-old *Ccr2*^−/−^ animals, whereas Tim-4^+^CD4^+^ macrophages predominated (Fig. 4, C and D).

Using the gut shield chimera approach, we once again looked at replenishment from BM monocytes in adult animals (Fig. 4 E). At 7 wk after irradiation, frequencies and numbers of all macrophage subsets in chimeric animals were indistinguishable from nonirradiated controls (Fig. S3 A). Importantly, between 7–16 wk after irradiation, Tim-4^+^CD4^+^ macrophages were not replenished from circulating precursors (Fig. 4 F), demonstrating that in the colon, this population is locally maintained. Tim-4^–^ macrophages were replenished from circulating precursors, but Tim-4^–^CD4^+^ macrophages achieved only 30% of their potential replenishment by 16 wk (Fig. 4 F), again supporting the fact that this population is replenished slowly from monocytes, whereas the Tim-4^–^CD4^–^ population is the only rapidly replenished population. Interestingly, differences in rates of monocyte replenishment were evident between the small intestine and colon (Fig. S3 B). Of particular note, Tim-4^–^CD4^+^ macrophages were replenished substantially more slowly from monocytes in the colon than in the small intestine (Fig. S3 B). Distinct tissue architecture (Mowat and Agace, 2014), exposure to specific bacteria or dietary-derived factors, and precise localization of the macrophage subsets between the colon and small intestine could all potentially underlie these variations in monocyte replenishment. These factors could similarly underlie the observed differences between colon and small intestine macrophage subset development early after birth.

Combined, these data demonstrate that even in the commensal-rich colon, Tim-4 delineates a population of previously unappreciated resident macrophages maintained independently of blood monocytes. Moreover, a substantial proportion of the monocyte-derived macrophages turn over at a slow, rather than rapid, rate from blood monocytes.

Based on our new understanding of macrophage development in the colon, we finally investigated the impact of commensals on both monocyte-derived and tissue-resident macrophage populations. In tissues including the skin (Tamoutounour et al., 2013) and peritoneal cavity (Kim et al., 2016), commensals selectively support monocyte-replenished macrophage populations. Corroborating published data (Rivollier et al., 2012; Bain et al., 2014), total monocytes and macrophages in the colon were decreased in adult germ-free (GF) animals (9 wk) when compared with specific pathogen–free (SPF) controls maintained in the same facility (Fig. S3 C). Unexpectedly, upon analysis of the new macrophage subsets, there were no striking alterations in the frequency of Tim-4^–^ monocyte–replenished (Tim-4^–^CD4^+^ and CD4^–^) populations compared with resident Tim-4^+^ macrophage subsets in GF intestine (Fig. 4 G). In line with this finding, absolute numbers were decreased in all populations of gut macrophages irrespective of their ontogeny (Fig. 4 H). Thus, a live commensal microbiome plays an important role in regulating the total number of macrophages in the colon irrespective of whether they are locally maintained or monocyte-replenished subsets.

Overall, our study presents a major extension to our understanding of resident gut macrophage replenishment in the mouse small intestine and colon to include both short-lived monocyte-dependent and long-lived monocyte-independent macrophage subsets. Previous studies using strategies including transgenic reporter animals, parabiosis, and monocyte transfer concluded that gut macrophages are BM monocyte derived (Jung et al., 2000; Bogunovic et al., 2009; Varol et al., 2009; Tamoutounour et al., 2012; Zigmond et al., 2012; Bain et al., 2013, 2014; Sheng et al., 2015). Our refined model does not disagree with these studies as we also find that >60% of the macrophages are BM monocyte derived, albeit ∼30% at a much slower rate than was previously suggested (Jaensson et al., 2008). One likely explanation for the locally maintained population being previously overlooked in these studies is an inability to discriminate this population without phenotypic markers from the high monocyte turnover population. Another factor that is interesting to speculate could have additionally confounded the identification of the long-lived macrophages, particularly using lineage-tracing animals (Sheng et al., 2015), is the perinatal ontogeny of these macrophages. Although it has been reported (Bain et al., 2014) using Ki67 staining that gut macrophages expand locally in the first few weeks after birth, it is possible that BM monocytes could be incorporated into the pool specifically at these early life time points. This has recently been demonstrated for Tim-4^+^ Kupffer cells that, although maintained locally and independent of blood monocytes in the adult, could incorporate monocytes during the rapid perinatal growth period of this organ (Scott et al., 2016). Whether early life expansion of Tim-4^+^CD4^+^ gut macrophages during intestinal development could also be supported by a temporally restricted wave of circulating monocytes entering the tissue will require further detailed experimental analysis.

This revised paradigm of mouse gut macrophage development bears striking similarity to a recent study in duodenal transplant patients (Bujko et al., 2018). In this study, alongside high–monocyte turnover macrophage populations, distinct and dominant subsets of slow-turnover populations were also reported. Irrespective of the precise correlations between mouse and human subsets combined with our data, this demonstrates that across species, the view of the gut as a site of persistent rapid monocyte-to-macrophage transition must be reconsidered.

The significance of a Tim-4–associated module of gene expression on a gut macrophage subset in relation to their activity has not yet been determined. However, given that Tim-4 is an important apoptotic cell uptake receptor (Miyanishi et al., 2007; Kuchroo et al., 2008; Nishi et al., 2014) and that Tim-4^+^ cells also express other gene transcripts associated with this process, e.g., *Edil3* and *P2ry2* (Fig. 1 I), a likely possibility is that they are more specialized in performing efferocytosis. This is concordant with their long tissue residency as recent studies have highlighted the importance of this function by resident macrophage populations (Uderhardt et al., 2012; Cummings et al., 2016; A-Gonzalez et al., 2017) including those in the T cell zone of lymph nodes as well as germinal centers of Peyer’s patches (Bonnardel et al., 2015; Baratin et al., 2017).

Independent of their functional heterogeneity or ontogeny, future studies of gut macrophage activity should be based on this new understanding that locally maintained and slow–monocyte turnover macrophages are abundantly present in the gut. In particular, our findings raise the previously unappreciated possibility of therapeutically manipulating influxing monocytes while keeping a protective tissue-resident population intact in life-limiting disease settings such as inflammatory bowel disease.

## Materials and methods

### Mice

C57BL/6J mice (CD45.2) were purchased from Envigo and housed in individually ventilated cages under SPF conditions. *Ccr2*^−/−^ animals (originally from The Jackson Laboratory; Boring et al., 1997), *Cx3cr1*^+/GFP^ (available from The Jackson Laboratory and provided by S. Jung; Jung et al., 2000), and congenic CD45.1 mice (serially backcrossed from SJL/J onto C57BL/6) were all bred in-house and shared by K. Else, K. Couper, and A. MacDonald (University of Manchester, Manchester, England, UK), respectively, and were backcrossed to a C57BL/6 background for at least 10 generations. *Cx3cr1*^CreER^ mice (from The Jackson Laboratory; Yona et al., 2013) were bred in-house crossed with *R26-yfp* mice (from The Jackson Laboratory; Srinivas et al., 2001) and were backcrossed with C57BL/6J for at least six generations. For GF experiments, all mice including SPF controls were bred in-house and were on a C57BL/6 background. GF C57BL/6 mice (founders from the Clean Mouse Facility, University of Bern, Bern, Switzerland) were bred and maintained in The University of Manchester gnotobiotic facility. SPF controls were also housed and maintained on the same diet and light cycle as GF animals. All experiments were approved by The University of Manchester Local Ethical Review Committee and were performed in accordance with the UK Home Office Animals (Scientific Procedures) Act 1986.

### Generation of shield chimeras

WT CD45.2^+^ host mice aged 6–8 wk were anaesthetized by intraperitoneal administration of ketamine (80 mg/kg; Vetoquinol) and xylazine (8 mg/kg; Bayer). Anaesthetized mice were positioned beneath a lead sheet shielding the lower two thirds of the body, including the intestine, from a split dose of irradiation (2× 5.5 Gy). Mice therefore received partial body irradiation with only the head, thorax, and forelimbs left exposed. After recovery from anesthesia, mice were reconstituted by intravenous injection with 2 × 10^6^ CD90.2^+^ T cell–depleted donor BM cells from congenic CD45.1^+^ WT donor animals. T cells were depleted using CD90.2 microbeads (Miltenyi Biotec). Mice were maintained on 0.03% enrofloxacin in drinking water for up to 1 wk before and for 2 wk after irradiation and then were housed in autoclaved cages with sterile water, diet, and bedding. Reconstitution was allowed to occur for a minimum of 7 wk before analysis.

### Tamoxifen treatment

Tamoxifen (Sigma-Aldrich) was dissolved in 10% ethanol and 90% corn oil to a concentration of 50 mg/ml. Mice were dosed with 5 mg by oral gavage for five consecutive days.

### Tissue preparation and cell isolation

#### Small intestine and colon lamina propria and muscularis

Cells were isolated as previously described with some modifications (Sun et al., 2007). In brief, after dissection of the small intestine and colon, Peyer’s patches were removed from the length of the small intestine, and both small intestine and colon were cut longitudinally and washed thoroughly with PBS on ice. Subsequently, to remove intestinal epithelial cells and leukocytes, small intestines and colons were cut into segments (2–3 cm) and incubated in prewarmed media (RPMI 1640) supplemented with 3% FCS, 20 mM Hepes, 100 U/ml polymyxin B (Sigma-Aldrich), 5 mM EDTA, and 1 mM freshly thawed dithiothreitol for 20 min at 37°C with agitation. After incubation, gut segments were repeatedly shaken in fresh serum-free media with 2 mM EDTA and 20 mM Hepes to ensure optimal dissociation of intestinal epithelial cells and leukocytes. Remaining tissue (lamina propria and muscularis) was minced and digested at 37°C for 30 min with continuous stirring in serum-free RPMI containing 20 mM Hepes, 0.1 mg/ml liberase TL (Roche), and 0.5 mg/ml DNase. Digested tissue was passed sequentially through a 70-µm filter and 40-µm cell strainer, and after pelleting, it was resuspended in media supplemented with 10% FCS and polymyxin B until staining.

#### Blood

Blood was collected into EDTA-coated syringes by cardiac puncture from sacrificed mice. Suspensions were washed and resuspended in ammonium-chloride-potassium lysing buffer (Lonza) for 3 min on ice twice. Suspensions were then washed and resuspended in media containing 10% FCS until staining.

#### Brain

Cells were isolated as previously described but with some modifications (Legroux et al., 2015). In brief, brains were minced and digested in media containing 20 mM Hepes, 2 mg/ml collagenase IV (Thermo Fisher Scientific), and 0.5 mg/ml DNase for 30 min at 37°C without agitation. Brains were subsequently passed through a 70-µm filter, washed with media supplemented with 20 mM Hepes and 2 mM EDTA, and myelin contaminants were removed over a 30% Percoll (Sigma-Aldrich) gradient, thus removing myelin and enriching for immune cells. The final pellet was resuspended in media containing 10% FCS until staining.

### Flow cytometry

Single-cell suspensions (5 × 10^5^–2 × 10^6^ total cells) of the small intestine, colon, or blood and the majority of cells isolated from brain tissue were washed thoroughly with PBS and stained with the Live/Dead Fixable blue dead cell stain kit (Molecular Probes) to exclude dead cells. Subsequently, cells were stained in the dark for 15 min at 4°C with fluorochrome- or biotin-conjugated antibodies in PBS containing anti-CD16/CD32 (2.4G2; BioXcell). Cells were washed and, where necessary, incubated for a further 10 min with fluorochrome-conjugated streptavidin and then washed. In some cases, cells were immediately acquired live, or alternatively, after further washing, cells were fixed in 2% paraformaldehyde (Sigma-Aldrich) for 10 min at room temperature and ultimately resuspended in PBS before acquisition. Cells were stained with CD4 (RM4-5), CD11b (M1/70), CD11c (N418), CD45 (30F11), CD45.1 (A20), CD45.2 (104), CD51 (RMV-7), CD64 (X54-5/7.1), CD115 (AFS98), MHCII (I-A/I-E; M5/114.15.2), and Tim-4 (RMT4-54 and F31-5G3) from BioLegend as well as Ly6C (HK1.4) from eBioscience. The lineage antibody cocktail for excluding lymphocytes and granulocytes included Siglec F (E50-2440) from BD and TCRβ (H57-597), B220 (RA3-6B2), and Ly6G (1A8) from BioLegend. Cell acquisition was performed on an LSR Fortessa running FACSDIVA 8 software (BD). For each intestinal sample, typically 10,000–20,000 macrophages were collected. In the case of blood samples, typically 10,000 monocytes were collected. Data were analyzed using FlowJo software (TreeStar).

### Gut monocyte and macrophage isolation by FACS

Single-cell suspensions for gut were prepared as above with the following modifications: incubation with dithiothreitol and EDTA was reduced to 10 min, and the liberase digestion step was decreased to 20 min but with an increased concentration of liberase TL (0.75 mg/ml). Before FACS, on a FACSAria Fusion (BD), isolated cells were suspended in RPMI supplemented with 2% FCS, 100 U/ml polymyxin B, and 2 mM EDTA. Cells were sorted using the same gating as in Fig. S1 A but with two modifications: cells were not initially gated based on CD11c expression, and Ly6C was included in the lineage channel to directly exclude P1 monocytes. Sorted cells were collected in RPMI with 10% FCS and stored on ice for use in cytospins and cell purity assessments or were alternatively collected in RLT buffer (QIAGEN) and stored on dry ice before storage at −80°C for subsequent RNA extraction.

### Cytospin

Sorted cells were mounted on superfrost slides using a Cytospin centrifuge (Cytospin 4; Thermo Fisher Scientific) operating for 5 min at 500 rpm. Cells were fixed with ice-cold methanol and stored at room temperature. Cells were subsequently stained with hematoxylin and acidic eosin and mounted with DPX. Images were collected on an Axioskop upright microscope (ZEISS) using a 100× objective and captured using a CoolSNAP ES camera (Photometrics) through MetaVue software (Molecular Devices). Images were then analyzed and processed using ImageJ (National Institutes of Health) and Image-Pro Premier software (Media Cybernetics).

### RNA extraction

RNA was extracted from 120,000–200,000 cells using an RNeasy micro kit (QIAGEN) following the manufacturer’s instructions. RNA was quantified using a Qubit 2.0 Fluorimeter (Thermo Fisher Scientific), and quality was assessed using an RNA ScreenTape Assay and 2200 TapeStation (Agilent Technologies).

### Bulk RNA sequencing and analysis

Strand-specific RNA sequencing libraries were prepared using the Illumina workflow with the TruSeq stranded mRNA sample preparation kit. Paired-end reads (65 × 65 bp) were generated from each sample. 48–192 million reads were obtained from each sample. The fastq files generated by a HiSeq4000 platform (Illumina) were analyzed with FastQC (http://www.bioinformatics.babraham.ac.uk/projects/fastqc/), and any low-quality reads and contaminated barcodes were trimmed with Trimmomactic (Bolger et al., 2014). All libraries were aligned to GRCm38.p4 assembly of mouse genome using STAR-2.4.2 (Dobin et al., 2013), and only uniquely mapped reads were used in differential gene expression analysis. The mapped reads were counted by genes with HTseq (Anders et al., 2015) against gencode.vM11.annotation.gtf. The differentially expressed genes were identified using DESeq2 (Love et al., 2014) by pairwise comparisons between the experimental groups. The differentially expressed genes with a p-adjusted value ≤ 0.05 were selected for further validation and analysis. For functional analysis, an R package of topGO, Ingenuity Pathway Analysis, and Panther was used. Bulk RNA sequencing data were deposited in the Gene Expression Omnibus public database under accession no. GSE114434.

### Cytometric bead array

Macrophage subsets isolated by FACS were plated in flat-bottomed 96-well tissue culture plates at 30,000 cells per well (two to three wells per subset). The cells were cultured at 37°C and 5% CO_2_ in 50 µl complete RPMI (10% FCS) in the presence of M-CSF (PeproTech) at 20 ng/ml. After 18 h, the culture supernatants were collected and analyzed for the presence of cytokines and chemokines using the LEGENDplex mouse inflammation panel (BioLegend). Data were acquired on a FACSVerse (BD) and analyzed using LEGENDplex software (7.1; BioLegend).

### Statistical analysis

Comparisons between groups were undertaken using Prism (7.0; GraphPad Software). Two experimental groups were compared using a Student’s *t* test for paired data or a Student’s *t* test with Welch’s correction for unpaired data. Where more than two groups were compared, a one-way ANOVA with Bonferroni’s correction was used. Significance was set at P ≤ 0.05.

### Online supplemental material

Fig. S1 shows the flow cytometry gating strategy for monocytes and macrophages in the small intestine, the PCA plot of global gene expression from Tim-4^–^CD4^–^–, Tim-4^–^CD4^+^–, and Tim-4^+^CD4^+^–resident macrophages, and the graphical representation of gene expression profiles for the 12 clusters formed by the 2,283 genes differentially expressed in Ly6C^hi^ blood monocytes and Tim-4^–^CD4^–^, Tim-4^–^CD4^+^, and Tim-4^+^CD4^+^ macrophages from the small intestine. Fig. S2 shows the total number of macrophages and number of Tim-4^–^CD4^–^, Tim-4^–^CD4^+^, and Tim-4^+^CD4^+^ macrophages in the small intestine of gut-shielded irradiated chimeric mice and unirradiated mice, the time course of tamoxifen treatment and harvest of tamoxifen-treated mice, and the YFP expression in the Ly6C^low^ blood monocytes and microglia of tamoxifen-treated mice. Fig. S3 shows the total number of macrophages and number of Tim-4^–^CD4^–^, Tim-4^–^CD4^+^, and Tim-4^+^CD4^+^ macrophages in the colon of gut-shielded irradiated chimeric mice and unirradiated mice, the comparison of chimerism levels reached in the macrophage subsets from the small intestine and colon, and the number of P1 monocytes, P2 transitioning monocytes, and P3/P4 total macrophages in the colon of SPF and GF mice. Table S1 shows the GO terms associated with each of the 12 clusters formed by the 2,283 genes differentially expressed in Ly6C^hi^ blood monocytes and Tim-4^–^CD4^–^, Tim-4^–^CD4^+^, and Tim-4^+^CD4^+^ macrophages sorted from the small intestine. Table S2 shows the list of genes contained in each of the 12 clusters formed by the 2,283 genes differentially expressed in Ly6C^hi^ blood monocytes and Tim-4^–^CD4^–^, Tim-4^–^CD4^+^, and Tim-4^+^CD4^+^ macrophages sorted from the small intestine.

## Supplementary Material

Supplemental Materials (PDF)

## References

[bib1] Afik R., Zigmond E., Vugman M., Klepfish M., Shimshoni E., Pasmanik-Chor M., Shenoy A., Bassat E., Halpern Z., Geiger T., 2016 Tumor macrophages are pivotal constructors of tumor collagenous matrix. J. Exp. Med. 213:2315–2331. 10.1084/jem.20151193 27697834 PMC5068227

[bib2] A-Gonzalez N., Quintana J.A., García-Silva S., Mazariegos M., González de la Aleja A., Nicolás-Ávila J.A., Walter W., Adrover J.M., Crainiciuc G., Kuchroo V.K., 2017 Phagocytosis imprints heterogeneity in tissue-resident macrophages. J. Exp. Med. 214:1281–1296. 10.1084/jem.20161375 28432199 PMC5413334

[bib3] Anders S., Pyl P.T., and Huber W. 2015 HTSeq--a Python framework to work with high-throughput sequencing data. Bioinformatics. 31:166–169. 10.1093/bioinformatics/btu638 25260700 PMC4287950

[bib4] Bain C.C., and Mowat A.M. 2014 Macrophages in intestinal homeostasis and inflammation. Immunol. Rev. 260:102–117. 10.1111/imr.12192 24942685 PMC4141699

[bib5] Bain C.C., Scott C.L., Uronen-Hansson H., Gudjonsson S., Jansson O., Grip O., Guilliams M., Malissen B., Agace W.W., and Mowat A.M. 2013 Resident and pro-inflammatory macrophages in the colon represent alternative context-dependent fates of the same Ly6Chi monocyte precursors. Mucosal Immunol. 6:498–510. 10.1038/mi.2012.89 22990622 PMC3629381

[bib6] Bain C.C., Bravo-Blas A., Scott C.L., Perdiguero E.G., Geissmann F., Henri S., Malissen B., Osborne L.C., Artis D., and Mowat A.M. 2014 Constant replenishment from circulating monocytes maintains the macrophage pool in the intestine of adult mice. Nat. Immunol. 15:929–937. 10.1038/ni.2967 25151491 PMC4169290

[bib7] Bain C.C., Hawley C.A., Garner H., Scott C.L., Schridde A., Steers N.J., Mack M., Joshi A., Guilliams M., Mowat A.M., 2016 Long-lived self-renewing bone marrow-derived macrophages displace embryo-derived cells to inhabit adult serous cavities. Nat. Commun. 7:s11852. 10.1038/ncomms11852 PMC4910019 27292029

[bib8] Baratin M., Simon L., Jorquera A., Ghigo C., Dembele D., Nowak J., Gentek R., Wienert S., Klauschen F., Malissen B., 2017 T Cell Zone Resident Macrophages Silently Dispose of Apoptotic Cells in the Lymph Node. Immunity. 47:349–362.e5. 28801233 10.1016/j.immuni.2017.07.019

[bib9] Bogunovic M., Ginhoux F., Helft J., Shang L., Hashimoto D., Greter M., Liu K., Jakubzick C., Ingersoll M.A., Leboeuf M., 2009 Origin of the lamina propria dendritic cell network. Immunity. 31:513–525. 10.1016/j.immuni.2009.08.010 19733489 PMC2778256

[bib10] Bolger A.M., Lohse M., and Usadel B. 2014 Trimmomatic: a flexible trimmer for Illumina sequence data. Bioinformatics. 30:2114–2120. 10.1093/bioinformatics/btu170 24695404 PMC4103590

[bib11] Bonnardel J., Da Silva C., Henri S., Tamoutounour S., Chasson L., Montañana-Sanchis F., Gorvel J.P., and Lelouard H. 2015 Innate and adaptive immune functions of peyer’s patch monocyte-derived cells. Cell Reports. 11:770–784. 10.1016/j.celrep.2015.03.067 25921539

[bib12] Boring L., Gosling J., Chensue S.W., Kunkel S.L., Farese R.V. Jr., Broxmeyer H.E., and Charo I.F. 1997 Impaired monocyte migration and reduced type 1 (Th1) cytokine responses in C-C chemokine receptor 2 knockout mice. J. Clin. Invest. 100:2552–2561. 10.1172/JCI119798 9366570 PMC508456

[bib13] Bujko A., Atlasy N., Landsverk O.J.B., Richter L., Yaqub S., Horneland R., Øyen O., Aandahl E.M., Aabakken L., Stunnenberg H.G., 2018 Transcriptional and functional profiling defines human small intestinal macrophage subsets. J. Exp. Med. 215:441–458. 29273642 10.1084/jem.20170057 PMC5789404

[bib14] Cummings R.J., Barbet G., Bongers G., Hartmann B.M., Gettler K., Muniz L., Furtado G.C., Cho J., Lira S.A., and Blander J.M. 2016 Different tissue phagocytes sample apoptotic cells to direct distinct homeostasis programs. Nature. 539:565–569. 10.1038/nature20138 27828940 PMC5807003

[bib15] Dobin A., Davis C.A., Schlesinger F., Drenkow J., Zaleski C., Jha S., Batut P., Chaisson M., and Gingeras T.R. 2013 STAR: ultrafast universal RNA-seq aligner. Bioinformatics. 29:15–21. 10.1093/bioinformatics/bts635 23104886 PMC3530905

[bib16] Gabanyi I., Muller P.A., Feighery L., Oliveira T.Y., Costa-Pinto F.A., and Mucida D. 2016 Neuro-immune Interactions Drive Tissue Programming in Intestinal Macrophages. Cell. 164:378–391. 10.1016/j.cell.2015.12.023 26777404 PMC4733406

[bib17] Gautier E.L., Shay T., Miller J., Greter M., Jakubzick C., Ivanov S., Helft J., Chow A., Elpek K.G., Gordonov S., Immunological Genome Consortium 2012 Gene-expression profiles and transcriptional regulatory pathways that underlie the identity and diversity of mouse tissue macrophages. Nat. Immunol. 13:1118–1128. 10.1038/ni.2419 23023392 PMC3558276

[bib18] Ginhoux F., and Guilliams M. 2016 Tissue-Resident Macrophage Ontogeny and Homeostasis. Immunity. 44:439–449. 10.1016/j.immuni.2016.02.024 26982352

[bib19] Ginhoux F., and Jung S. 2014 Monocytes and macrophages: developmental pathways and tissue homeostasis. Nat. Rev. Immunol. 14:392–404. 10.1038/nri3671 24854589

[bib20] Ginhoux F., Greter M., Leboeuf M., Nandi S., See P., Gokhan S., Mehler M.F., Conway S.J., Ng L.G., Stanley E.R., 2010 Fate mapping analysis reveals that adult microglia derive from primitive macrophages. Science. 330:841–845. 10.1126/science.1194637 20966214 PMC3719181

[bib21] Goldmann T., Wieghofer P., Jordão M.J., Prutek F., Hagemeyer N., Frenzel K., Amann L., Staszewski O., Kierdorf K., Krueger M., 2016 Origin, fate and dynamics of macrophages at central nervous system interfaces. Nat. Immunol. 17:797–805. 10.1038/ni.3423 27135602 PMC4968048

[bib22] Grainger J.R., Konkel J.E., Zangerle-Murray T., and Shaw T.N. 2017 Macrophages in gastrointestinal homeostasis and inflammation. Pflugers Arch. 469:527–539. 10.1007/s00424-017-1958-2 28283748 PMC5362667

[bib23] Gross M., Salame T.M., and Jung S. 2015 Guardians of the Gut - Murine Intestinal Macrophages and Dendritic Cells. Front. Immunol. 6:254 10.3389/fimmu.2015.00254 26082775 PMC4451680

[bib24] Guilliams M., De Kleer I., Henri S., Post S., Vanhoutte L., De Prijck S., Deswarte K., Malissen B., Hammad H., and Lambrecht B.N. 2013 Alveolar macrophages develop from fetal monocytes that differentiate into long-lived cells in the first week of life via GM-CSF. J. Exp. Med. 210:1977–1992. 10.1084/jem.20131199 24043763 PMC3782041

[bib25] Hashimoto D., Chow A., Noizat C., Teo P., Beasley M.B., Leboeuf M., Becker C.D., See P., Price J., Lucas D., 2013 Tissue-resident macrophages self-maintain locally throughout adult life with minimal contribution from circulating monocytes. Immunity. 38:792–804. 10.1016/j.immuni.2013.04.004 23601688 PMC3853406

[bib26] Hoeffel G., Wang Y., Greter M., See P., Teo P., Malleret B., Leboeuf M., Low D., Oller G., Almeida F., 2012 Adult Langerhans cells derive predominantly from embryonic fetal liver monocytes with a minor contribution of yolk sac-derived macrophages. J. Exp. Med. 209:1167–1181. 10.1084/jem.20120340 22565823 PMC3371735

[bib27] Hoeffel G., Chen J., Lavin Y., Low D., Almeida F.F., See P., Beaudin A.E., Lum J., Low I., Forsberg E.C., 2015 C-Myb(+) erythro-myeloid progenitor-derived fetal monocytes give rise to adult tissue-resident macrophages. Immunity. 42:665–678. 10.1016/j.immuni.2015.03.011 25902481 PMC4545768

[bib28] Jaensson E., Uronen-Hansson H., Pabst O., Eksteen B., Tian J., Coombes J.L., Berg P.L., Davidsson T., Powrie F., Johansson-Lindbom B., and Agace W.W. 2008 Small intestinal CD103+ dendritic cells display unique functional properties that are conserved between mice and humans. J. Exp. Med. 205:2139–2149. 10.1084/jem.20080414 18710932 PMC2526207

[bib29] Jung S., Aliberti J., Graemmel P., Sunshine M.J., Kreutzberg G.W., Sher A., and Littman D.R. 2000 Analysis of fractalkine receptor CX(3)CR1 function by targeted deletion and green fluorescent protein reporter gene insertion. Mol. Cell. Biol. 20:4106–4114. 10.1128/MCB.20.11.4106-4114.2000 10805752 PMC85780

[bib30] Kamada N., Hisamatsu T., Okamoto S., Chinen H., Kobayashi T., Sato T., Sakuraba A., Kitazume M.T., Sugita A., Koganei K., 2008 Unique CD14 intestinal macrophages contribute to the pathogenesis of Crohn disease via IL-23/IFN-gamma axis. J. Clin. Invest. 118:2269–2280. 18497880 10.1172/JCI34610 PMC2391067

[bib31] Kim K.W., Williams J.W., Wang Y.T., Ivanov S., Gilfillan S., Colonna M., Virgin H.W., Gautier E.L., and Randolph G.J. 2016 MHC II+ resident peritoneal and pleural macrophages rely on IRF4 for development from circulating monocytes. J. Exp. Med. 213:1951–1959. 10.1084/jem.20160486 27551152 PMC5030807

[bib32] Kuchroo V.K., Dardalhon V., Xiao S., and Anderson A.C. 2008 New roles for TIM family members in immune regulation. Nat. Rev. Immunol. 8:577–580. 10.1038/nri2366 18617884

[bib33] Legroux L., Pittet C.L., Beauseigle D., Deblois G., Prat A., and Arbour N. 2015 An optimized method to process mouse CNS to simultaneously analyze neural cells and leukocytes by flow cytometry. J. Neurosci. Methods. 247:23–31. 10.1016/j.jneumeth.2015.03.021 25819540

[bib34] Loke P., Nair M.G., Parkinson J., Guiliano D., Blaxter M., and Allen J.E. 2002 IL-4 dependent alternatively-activated macrophages have a distinctive in vivo gene expression phenotype. BMC Immunol. 3:7 10.1186/1471-2172-3-7 12098359 PMC117781

[bib35] Love M.I., Huber W., and Anders S. 2014 Moderated estimation of fold change and dispersion for RNA-seq data with DESeq2. Genome Biol. 15:550 10.1186/s13059-014-0550-8 25516281 PMC4302049

[bib36] Merad M., Manz M.G., Karsunky H., Wagers A., Peters W., Charo I., Weissman I.L., Cyster J.G., and Engleman E.G. 2002 Langerhans cells renew in the skin throughout life under steady-state conditions. Nat. Immunol. 3:1135–1141. 10.1038/ni852 12415265 PMC4727838

[bib37] Miyanishi M., Tada K., Koike M., Uchiyama Y., Kitamura T., and Nagata S. 2007 Identification of Tim4 as a phosphatidylserine receptor. Nature. 450:435–439. 10.1038/nature06307 17960135

[bib38] Mowat A.M., and Agace W.W. 2014 Regional specialization within the intestinal immune system. Nat. Rev. Immunol. 14:667–685. 10.1038/nri3738 25234148

[bib39] Muller P.A., Koscsó B., Rajani G.M., Stevanovic K., Berres M.L., Hashimoto D., Mortha A., Leboeuf M., Li X.M., Mucida D., 2014 Crosstalk between muscularis macrophages and enteric neurons regulates gastrointestinal motility. Cell. 158:300–313. 10.1016/j.cell.2014.04.050 25036630 PMC4149228

[bib40] Nakatsukasa H., Zhang D., Maruyama T., Chen H., Cui K., Ishikawa M., Deng L., Zanvit P., Tu E., Jin W., 2015 The DNA-binding inhibitor Id3 regulates IL-9 production in CD4(+) T cells. Nat. Immunol. 16:1077–1084. 10.1038/ni.3252 26322481 PMC5935106

[bib41] Nishi C., Toda S., Segawa K., and Nagata S. 2014 Tim4- and MerTK-mediated engulfment of apoptotic cells by mouse resident peritoneal macrophages. Mol. Cell. Biol. 34:1512–1520. 10.1128/MCB.01394-13 24515440 PMC3993587

[bib42] O’Hara A.M., and Shanahan F. 2006 The gut flora as a forgotten organ. EMBO Rep. 7:688–693. 10.1038/sj.embor.7400731 16819463 PMC1500832

[bib43] Pull S.L., Doherty J.M., Mills J.C., Gordon J.I., and Stappenbeck T.S. 2005 Activated macrophages are an adaptive element of the colonic epithelial progenitor niche necessary for regenerative responses to injury. Proc. Natl. Acad. Sci. USA. 102:99–104. 10.1073/pnas.0405979102 15615857 PMC544052

[bib44] Rivollier A., He J., Kole A., Valatas V., and Kelsall B.L. 2012 Inflammation switches the differentiation program of Ly6Chi monocytes from antiinflammatory macrophages to inflammatory dendritic cells in the colon. J. Exp. Med. 209:139–155. 10.1084/jem.20101387 22231304 PMC3260867

[bib45] Rosas M., Davies L.C., Giles P.J., Liao C.T., Kharfan B., Stone T.C., O’Donnell V.B., Fraser D.J., Jones S.A., and Taylor P.R. 2014 The transcription factor Gata6 links tissue macrophage phenotype and proliferative renewal. Science. 344:645–648. 10.1126/science.1251414 24762537 PMC4185421

[bib46] Schridde A., Bain C.C., Mayer J.U., Montgomery J., Pollet E., Denecke B., Milling S.W.F., Jenkins S.J., Dalod M., Henri S., 2017 Tissue-specific differentiation of colonic macrophages requires TGFβ receptor-mediated signaling. Mucosal Immunol. 10:1387–1399. 10.1038/mi.2016.142 28145440 PMC5417360

[bib47] Schulz C., Gomez Perdiguero E., Chorro L., Szabo-Rogers H., Cagnard N., Kierdorf K., Prinz M., Wu B., Jacobsen S.E., Pollard J.W., 2012 A lineage of myeloid cells independent of Myb and hematopoietic stem cells. Science. 336:86–90. 10.1126/science.1219179 22442384

[bib48] Scott C.L., Zheng F., De Baetselier P., Martens L., Saeys Y., De Prijck S., Lippens S., Abels C., Schoonooghe S., Raes G., 2016 Bone marrow-derived monocytes give rise to self-renewing and fully differentiated Kupffer cells. Nat. Commun. 7:10321 10.1038/ncomms10321 26813785 PMC4737801

[bib49] Serbina N.V., and Pamer E.G. 2006 Monocyte emigration from bone marrow during bacterial infection requires signals mediated by chemokine receptor CCR2. Nat. Immunol. 7:311–317. 10.1038/ni1309 16462739

[bib50] Sheng J., Ruedl C., and Karjalainen K. 2015 Most Tissue-Resident Macrophages Except Microglia Are Derived from Fetal Hematopoietic Stem Cells. Immunity. 43:382–393. 10.1016/j.immuni.2015.07.016 26287683

[bib51] Smythies L.E., Sellers M., Clements R.H., Mosteller-Barnum M., Meng G., Benjamin W.H., Orenstein J.M., and Smith P.D. 2005 Human intestinal macrophages display profound inflammatory anergy despite avid phagocytic and bacteriocidal activity. J. Clin. Invest. 115:66–75. 10.1172/JCI200519229 15630445 PMC539188

[bib52] Srinivas S., Watanabe T., Lin C.S., William C.M., Tanabe Y., Jessell T.M., and Costantini F. 2001 Cre reporter strains produced by targeted insertion of EYFP and ECFP into the ROSA26 locus. BMC Dev. Biol. 1:4 10.1186/1471-213X-1-4 11299042 PMC31338

[bib53] Sun C.M., Hall J.A., Blank R.B., Bouladoux N., Oukka M., Mora J.R., and Belkaid Y. 2007 Small intestine lamina propria dendritic cells promote de novo generation of Foxp3 T reg cells via retinoic acid. J. Exp. Med. 204:1775–1785. 10.1084/jem.20070602 17620362 PMC2118682

[bib54] Tamoutounour S., Henri S., Lelouard H., de Bovis B., de Haar C., van der Woude C.J., Woltman A.M., Reyal Y., Bonnet D., Sichien D., 2012 CD64 distinguishes macrophages from dendritic cells in the gut and reveals the Th1-inducing role of mesenteric lymph node macrophages during colitis. Eur. J. Immunol. 42:3150–3166. 10.1002/eji.201242847 22936024

[bib55] Tamoutounour S., Guilliams M., Montanana Sanchis F., Liu H., Terhorst D., Malosse C., Pollet E., Ardouin L., Luche H., Sanchez C., 2013 Origins and functional specialization of macrophages and of conventional and monocyte-derived dendritic cells in mouse skin. Immunity. 39:925–938. 10.1016/j.immuni.2013.10.004 24184057

[bib56] Uderhardt S., Herrmann M., Oskolkova O.V., Aschermann S., Bicker W., Ipseiz N., Sarter K., Frey B., Rothe T., Voll R., 2012 12/15-lipoxygenase orchestrates the clearance of apoptotic cells and maintains immunologic tolerance. Immunity. 36:834–846. 10.1016/j.immuni.2012.03.010 22503541

[bib57] van Furth R., Cohn Z.A., Hirsch J.G., Humphrey J.H., Spector W.G., and Langevoort H.L. 1972 The mononuclear phagocyte system: a new classification of macrophages, monocytes, and their precursor cells. Bull. World Health Organ. 46:845–852. 4538544 PMC2480884

[bib58] Varol C., Vallon-Eberhard A., Elinav E., Aychek T., Shapira Y., Luche H., Fehling H.J., Hardt W.D., Shakhar G., and Jung S. 2009 Intestinal lamina propria dendritic cell subsets have different origin and functions. Immunity. 31:502–512. 10.1016/j.immuni.2009.06.025 19733097

[bib59] Yona S., Kim K.W., Wolf Y., Mildner A., Varol D., Breker M., Strauss-Ayali D., Viukov S., Guilliams M., Misharin A., 2013 Fate mapping reveals origins and dynamics of monocytes and tissue macrophages under homeostasis. Immunity. 38:79–91. 10.1016/j.immuni.2012.12.001 23273845 PMC3908543

[bib60] Zigmond E., Varol C., Farache J., Elmaliah E., Satpathy A.T., Friedlander G., Mack M., Shpigel N., Boneca I.G., Murphy K.M., 2012 Ly6C hi monocytes in the inflamed colon give rise to proinflammatory effector cells and migratory antigen-presenting cells. Immunity. 37:1076–1090. 10.1016/j.immuni.2012.08.026 23219392

