# Supplementary material for: Tissue-resident macrophages in the intestine are long lived and defined by Tim-4 and CD4 expression
Source: J Exp Med. 2018 Jun 4;215(6):1507–18. doi: 10.1084/jem.20180019 (PMC5987925; doi:10.1084/jem.20180019)
Supplement: Supplemental Materials (PDF) [file JEM_20180019_sm.pdf]

Supplemental material

Shaw et al., https://doi.org/10.1084/jem.20180019

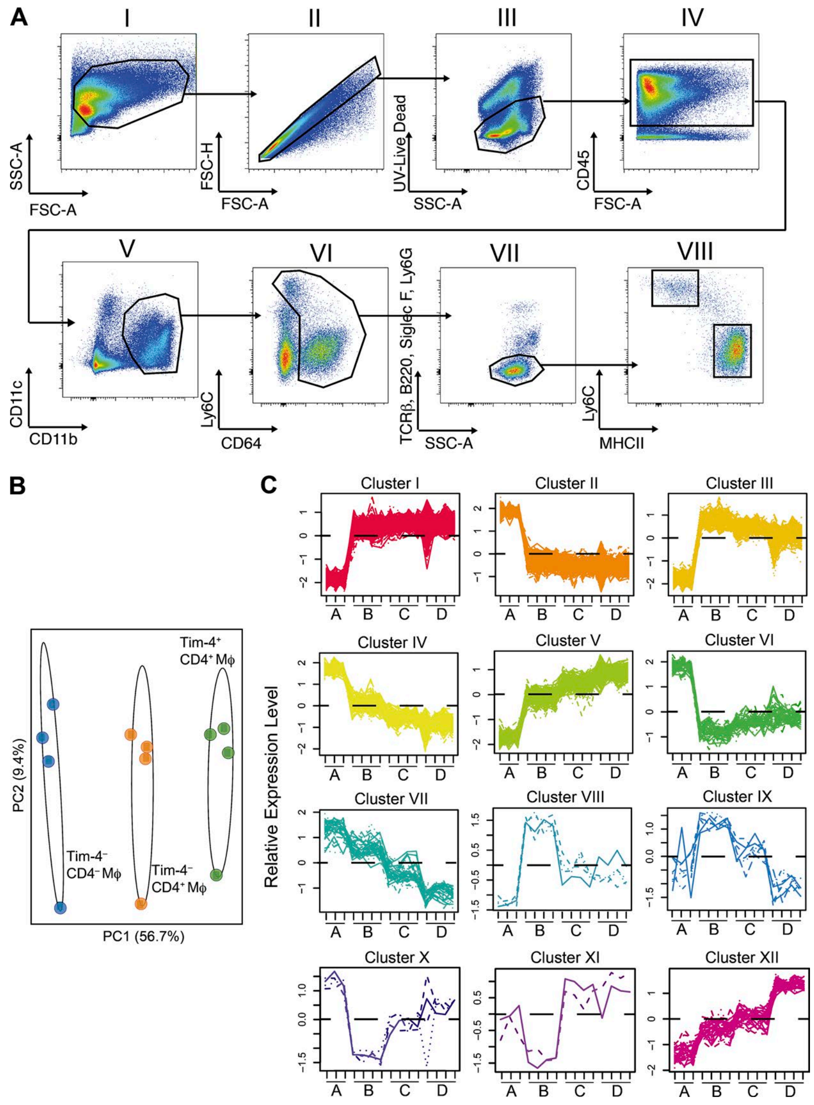

Figure S1. **Gating of gut macrophages and cluster analysis of Ly6C^{hi} blood monocytes and gut macrophages. (A)** Flow cytometry gating strategy for monocytes and macrophages in single-cell suspensions of combined lamina propria and muscularis. Cells were identified as follows: (I) Side scatter area (SSC-A) versus forward scatter area (FSC-A) to remove cellular debris; (II) forward scatter height (FSC-H) versus FSC-A for doublet exclusion; (III) Live-Dead versus SSC-A to identify live cells; (IV) CD45 versus FSC-A to define hematopoietic populations; (V and VI) CD11c versus CD11b and Ly6C versus CD64 to enrich for monocytes and macrophages; (VII) lineage versus SSC-A to exclude contaminating nonmonocyte and macrophage populations; and (VIII) Ly6C versus MHCII to visualize monocyte-to-macrophage waterfall. **(B)** PCA of global gene expression from Tim-4$^-$CD4$^-$, Tim-4$^-$CD4$^+$, and Tim-4$^+$CD4$^+$ resident macrophages isolated by FACS from the small intestine of 8–10-wk-old C57BL/6 WT mice. **(C)** Graphical representation of gene expression profiles for each cluster of differentially expressed genes established by k-means. Samples and sequencing were generated as described in Fig. 1 D. Cell populations: A, Ly6C^{hi} blood monocytes; B, Tim-4$^-$CD4$^-$ macrophages; C, Tim-4$^-$CD4$^+$ macrophages; D, Tim-4$^+$CD4$^+$ macrophages.

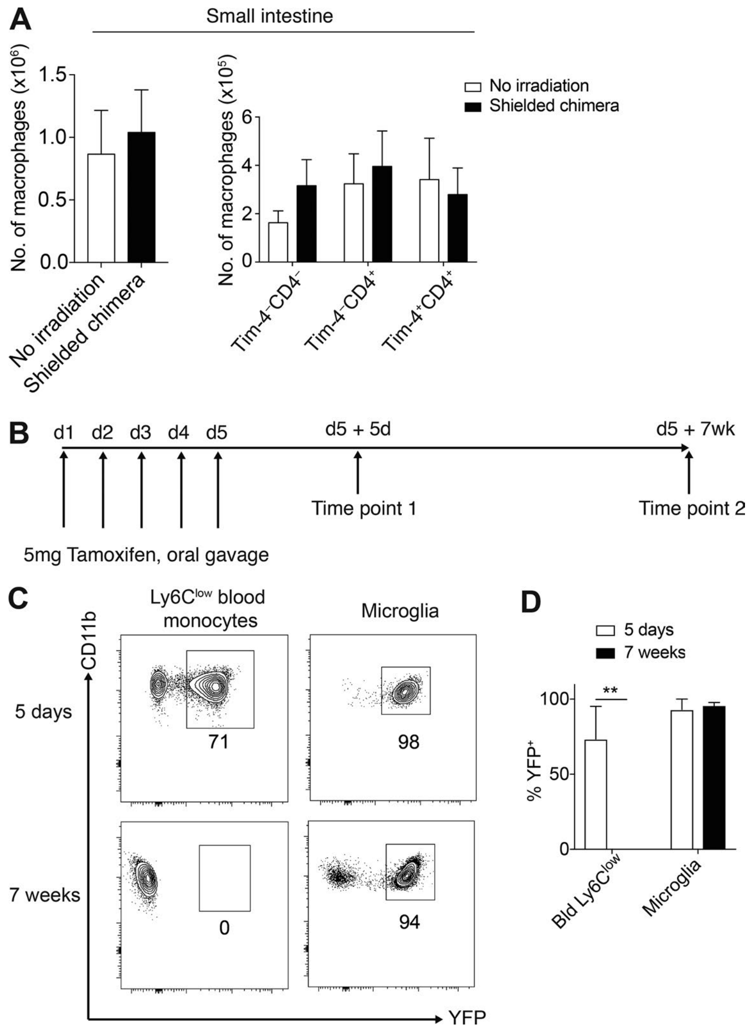

Figure S2. **Gut macrophage populations in gut-shield chimeras and YFP expression over time in Ly6C^{low} monocytes and microglia of *Cx3cr1CreER* x *R26-yfp* animals. (A)** Total number of macrophages and of Tim-4$^-$CD4$^-$, Tim-4$^-$CD4$^+$, and Tim-4$^+$CD4$^+$ macrophage subsets in small intestines of gut-shielded chimeras generated as described in Fig. 3 A at 7 wk after irradiation. Data are pooled from two experiments. *n* = 5–6 per group. **(B)** Schematic of tamoxifen administration and experimental endpoints in *Cx3cr1CreER*-crossed *R26-yfp* animals. **(C)** YFP reporter expression at 5 d and 7 wk after treatment with tamoxifen as shown in B in Ly6C^{low} blood monocytes and brain microglia. Numbers denote the percentages of cells within the gate. **(D)** Frequency of YFP$^+$ cells in Ly6C^{low} blood monocytes and brain microglia after treatment with tamoxifen as shown in B. Data are pooled from two independent experiments. *n* = 4–5 per group. Error bars show means ± SD. Statistical comparisons were performed with two-way Student's *t* test with Welch's correction: **, P ≤ 0.01.

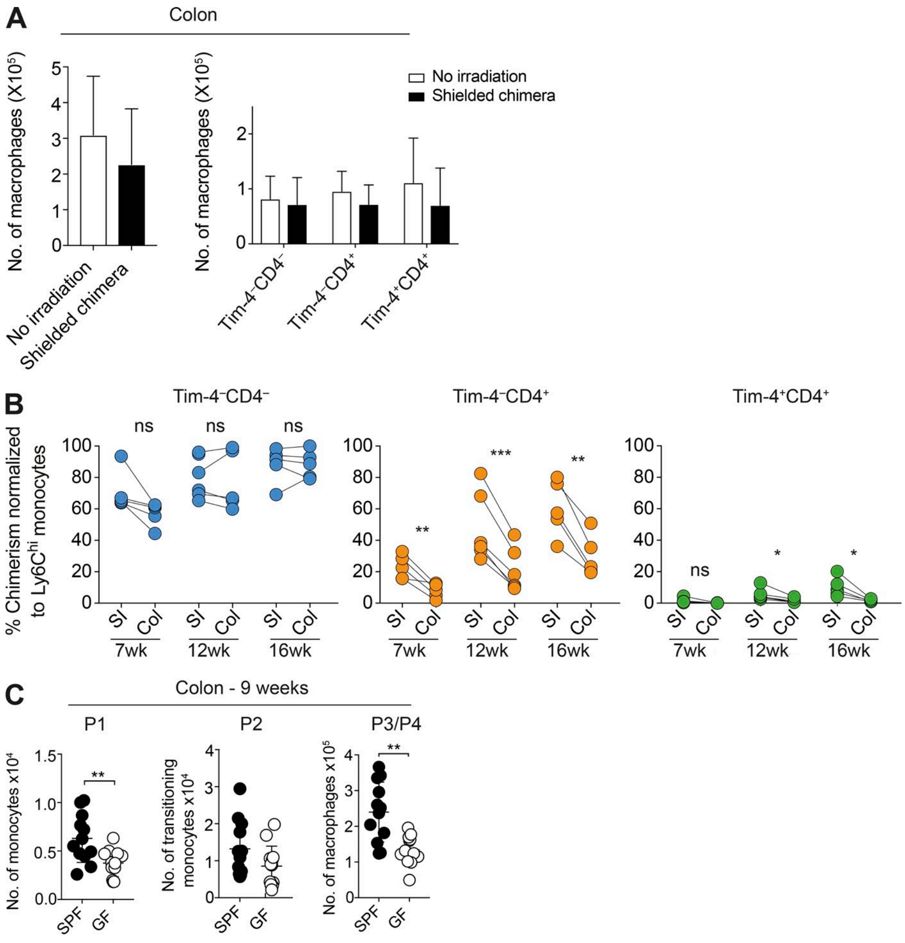

Figure S3. **Gut macrophage populations in gut-shield chimeras and reduced numbers of monocytes and macrophages in the colon of GF animals compared with SPF controls. (A)** Total number of macrophages and of Tim-4⁻CD4⁻, Tim-4⁻CD4⁺, and Tim-4⁺CD4⁺ macrophage subsets in the colon of gut-shielded chimeras generated as described in Fig. 3 A at 7 wk after irradiation. Data are pooled from two experiments. *n* = 5–6 per group. **(B)** At 7, 12, and 16 wk after irradiation, the frequency of donor-derived cells was determined in the intestinal Ly6C^{hi} monocyte and gut macrophage subpopulations of the small intestine (SI) and colon (Col) by flow cytometry and normalized to the chimerism of Ly6C^{hi} blood monocytes. Paired data from individual mice from at least two independent experiments are shown. *n* = 5 or 6 per group. Results for individual animals are shown as dots. **(C)** Total number of monocytes (P1), transitioning monocyte to macrophages cells (P2), and macrophages (P3/P4) within the colon of SPF and GF mice at 9 wk of age. Data are pooled from four independent experiments. *n* = 12 per group; results for individual animals are shown as dots. Error bars show means ± SD. Statistical comparisons were performed with two-way Student's *t* test with (C) or without (B) Welch's correction: *, P ≤ 0.05; **, P ≤ 0.01; ***, P ≤ 0.001.

Table S1. **GO terms by cluster**

Cluster	GO terms more than twofold enriched and P < 0.05
I	Protein ADP-ribosylation, cell growth, locomotion, response to abiotic stimulus, extracellular transport, negative regulation of apoptotic process, transmembrane receptor protein tyrosine kinase signaling pathway, phagocytosis, cell-cell adhesion, cytokine-mediated signaling pathway, cellular component movement, response to biotic stimulus, response to endogenous stimulus, B cell–mediated immunity, cell adhesion, biological adhesion, endocytosis, cell proliferation, MAPK cascade, and blood coagulation
II	Pentose-phosphate shunt, glycolysis, JNK cascade, lipid transport, I-κB kinase/NF-κB cascade, cellular defense response, negative regulation of apoptotic process, sensory perception of sound, calcium-mediated signaling, regulation of cell cycle, cytokinesis, intracellular signal transduction, cytokine-mediated signaling pathway, regulation of phosphate metabolic process, protein glycosylation, cellular amino acid metabolic process, and MAPK cascade
III	Antigen processing and presentation of peptide or polysaccharide antigen via MHC class II, antigen processing and presentation, DNA recombination, chromosome segregation, DNA replication, cellular defense response, meiosis, cell cycle, DNA repair, cytokinesis, macrophage activation, natural killer cell activation, lipid transport, regulation of cell cycle, immune system process, regulation of catalytic activity, regulation of molecular function, cytoskeleton organization, MAPK cascade, phosphate-containing compound metabolic process, cell adhesion, and biological adhesion
IV	Unsaturated fatty acid biosynthetic process, acyl-CoA metabolic process, neuromuscular synaptic transmission, neurotransmitter secretion, cation transport, coenzyme metabolic process, regulation of catalytic activity, cytokine-mediated signaling pathway, regulation of molecular function, cellular defense response, cell adhesion, biological adhesion, ion transport, MAPK cascade, B cell–mediated immunity, and regulation of phosphate metabolic process
V	Nitric oxide biosynthetic process, cyclic nucleotide metabolic process, cellular calcium ion homeostasis, homeostatic process, angiogenesis, locomotion, protein glycosylation, anatomical structure morphogenesis, regulation of molecular function, cell adhesion, biological adhesion, response to external stimulus, and cellular component movement
VI	Ferredoxin metabolic process, sensory perception of pain, asymmetric protein localization, antigen processing and presentation of peptide or polysaccharide antigen via MHC class II, antigen processing and presentation, purine nucleobase metabolic process, blood coagulation, cellular defense response, cell adhesion, biological adhesion, response to external stimulus, immune system process, and response to stress
VII	Regulation of vasoconstriction, phosphate ion transport, mammary gland development, cell–cell adhesion, blood circulation, phospholipid metabolic process, cell adhesion, biological adhesion, phosphate-containing compound metabolic process, and carbohydrate metabolic process
VIII	Hemopoiesis, blood coagulation, macrophage activation, immune system process, cell proliferation, cell–cell adhesion, and cellular defense response
IX	JAK-STAT cascade, JNK cascade, induction of apoptosis, I-κB kinase/NF-κB cascade, negative regulation of apoptotic process, hemopoiesis, cellular defense response, cytokine-mediated signaling pathway, MAPK cascade, cell adhesion, biological adhesion, apoptotic process, cell death, death, mesoderm development, intracellular signal transduction, immune response, phosphate-containing compound metabolic process, developmental process, signal transduction, cell communication, and cellular process
X	Not determined
XI	Hemopoiesis and B cell–mediated immunity
XII	Response to toxic substance, blood coagulation, response to external stimulus, cell–cell adhesion, biological adhesion, macrophage activation, immune system process, visual perception, and heart development

Table S2. **Gene lists by cluster**

Cluster	Genes
Cluster I	*1700016P03Rik, 1810011H11Rik, 2510009E07Rik, 2610528A11Rik, 2900026A02Rik, 3110043O21Rik, 4833411C07Rik, 4930427A07Rik, 4930503L19Rik, 4930539E08Rik, 4931406C07Rik, 5031425F14Rik, 6330403L08Rik, 9430076C15Rik, 9930120I10Rik, A530099J19Rik, A930006K02Rik, A930011G23Rik, Abcc3, Abcg2, Abcg3, Abhd6, Ablim1, Acp2, Acp5, Acsf2, Actn1, Acvrl1, Acy1, Adam19, Adam22, Adam23, Adamtsl4, Adap2, Adck5, Adgre1, Adgrg5, Adgrl3, Adora3, Agap1, Agmo, Ahrr, Aif1, Akr1e1, Aldh1a1, Aldob, Alox5, Amacr, Amica1, Anks6, Ano8, Anpep, Antxr1, Antxr2, Aoah, Ap1b1, Apoe, Apol10b, Apol7c, Apold1, Arfgef2, Arhgap18, Arhgap19, Arhgap32, Arhgap33, Arhgap35, Arhgef12, Arhgef17, Arl4c, Arpin, Arrb2, Art2a-ps, Art2b, As3mt, Asah2, Asb2, Asgr2, Atad5, Atp6ap2, AU020206, AW112010, Axl, B130021K23Rik, B230217C12Rik, B3galnt1, B4galt6, Baiap2, Bank1, Basp1, Batf3, BC026585, BC049352, BC055324, Bcat2, Bcl2a1a, Bcl2a1b, Bcl2a1d, Bend6, Bhlhe40, Bin1, Bmp1, Bmp2, Brca2, Btbd19, Btg2, Btnl2, C1qa, C1qb, C1qc, C1ra, C1s1, C3ar1, C5ar1, C5ar2, C6, Cables1, Cadm1, Cald1, Camk1, Camk2a, Capn3, Car13, Car2, Card11, Cav1, Cbarp, Cbr1, Cbx6, Ccdc102a, Ccdc134, Ccdc157, Ccl12, Ccl3, Ccl4, Ccl7, Ccl8, Ccnd2, Ccnl1, Ccr1, Ccr5, Cd164, Cd1d1, Cd200r4, Cd209e, Cd22, Cd274, Cd276, Cd33, Cd6, Cd63, Cd72, Cd79a, Cd79b, Cd80, Cd81, Cd82, Cd83, Cd86, Cd9, Cdc42bpg, Cdc7, Cdca5, Cdk5rap2, Cdkn1a, Cdr2, Cela1, Cenpi, Cep152, Cep57l1, Cep68, Cep89, Chd3, Chd7, Chka, Chp2, Chpf, Chst15, Chst7, Chtf18, Cish, Clasp2, Clec1b, Clec4n, Clec9a, Clic4, Clmp, Clspn, Cmah, Cmklr1, Cmtm4, Cmtm8, Cnrip1, Col14a1, Col15a1, Col18a1, Col1a1, Col3a1, Col4a1, Col4a2, Col5a1, Copz2, Cp, Cpeb4, Cpne2, Cpq, Creg2, Creld1, Crem, Crim1, Ctc1, Ctla2b, Ctsb, Ctsf, Ctsl, Ctso, Cttn, Cttnbp2nl, Cul7, Cxcl1, Cxcl10, Cxcl14, Cxcl16, Cxcl2, Cxx1c, D8Ertd82e, Dab2, Dclre1c, Ddit3, Ddx11, Ddx5, Decr2, Dennd1b, Derl3, Dgat1, Dhrs3, Dip2c, Dnaaf3, Dnajb2, Dnal1, Dnase1l3, Dnase2a, Dnmbp, Dock4, Dock6, Dock7, Dse, Dst, Dtl, Dtnbp1, Dtx4, Dusp1, Dusp8, Dynll2, Dyrk4, E2f1, Eef2k, Egr1, Ehbp1, Ehd1, Ehhadh, Enho, Enpp1, Enpp4, Enpp5, Entpd1, Eogt, Epb41l2, Ephx1, Ermap, Ets1, Ets2, Etv5, Eva1b, Exoc3l4, F11r, F2r, F3, F830016B08Rik, Fads1, Fads2, Fads3, Fam110b, Fam134b, Fam149a, Fam213a, Fam234b, Fam3c, Fam46c, Fblim1, Fbln1, Fcgrt, Fchsd2, Fcrlb, Fgd2, Fgf11, Fgfrl1, Fggy, Fgl2, Fhad1, Fhl1, Flnb, Fmn1, Fmnl2, Fmnl3, Fnbp1, Fos, Fosb, Frmd4b, Fscn1, Fth1, G530011O06Rik, Gaa, Gabbr1, Gadd45b, Gadd45g, Galnt12, Gas2l3, Gas6, Gatm, Gbgt1, Gbp2b, Gdf15, Gdpd1, Gdpd5, Gga2, Ggt5, Gimap1, Gimap3, Gimap4, Gimap6, Gla, Gm10134, Gm13431, Gm15344, Gm15523, Gm17024, Gm17971, Gm19434, Gm20056, Gm20186, Gm33280, Gm37347, Gm44250, Gm44292, Gm44861, Gm44950, Gm45043, Gm6377, Gna12, Gnaq, Gns, Got1, Gpr155, Gpr171, Gpr31b, Gpr34, Gpr55, Gpr65, Grap, Gsg1, Gstm5, Gtse1,H2-Oa, Hap1, Haus3, Hcar2, Hebp1, Herpud1, Hes1, Hexdc, Hfe, Hic1, Hilpda, Hlx, Hmces, Hmcn2, Hmgn1, Hmgn2, Hook2, Hpgds, Hpn, Hrh1, Hs2st1, Hs3st3b1, Hspa5, Htra1, Htra3, Hvcn1, Icosl, Idh2, Ier3, Ifi27, Igf1, Igfbp4, Igfbp7, Igha, Ighd, Ighj2, Ighv1-18, Ighv1-19, Ighv1-26, Ighv1-39, Ighv1-76, Ighv2-2, Ighv3-6, Igkc, Igkv1-110, Igkv10-96, Igkv5-43, Igkv5-48, Igkv6-23, Iglc1, Iglv1, Igsf8, Il10, Il10rb, Il11ra1, Il1bos, Il1r2, Il1rl2, Il4i1, Impact, Impdh2, Inpp4b, Inpp5j, Insig1, Insl6, Intu, Ipo13, Iqgap3, Irak2, Irf2bp2, Irs2, Ispd, Itga9, Itgax, Itgb5, Itih5, Itm2c, Itsn1, vns1abp, Jade2, Jag2, Jchain, Jun, Junb, Jund, Jup, Kank2, Kazn, Kcng2, Kcnip3, Kcnj10, Kcnk13, Kcnk6, Kctd12, Kdelc2, Kif13b, Kif19a, Kifap3, Kitl, Klrb1b, Kpnb1, Kynu, L2hgdh, Lacc1, Lag3, Lair1, Lamb2, Lclat1, Lgmn, Lilra5, Lix1l, Lmna, Loxl3, Lpar1, Lpar6, Lpcat2, Lrch1, Lrp12, Lrp6, Ltbp1, Ltc4s, Lzts2, Lzts3, Mab21l3, Maf, Mafb, Mag, Maged1, Maged2, Maml2, Man1a, Mansc1, March8, Marcks, Mbd4, Mcm8, Mdfic, Megf8, Meis3, Mertk, Mgat4a, Mgat5, Mgl2, Mical2, Mir5107, Mmd, Mmp13, Mmp14, Mmp2, Mmp9, Mpzl3, Mras, Mrc1, Mrvi1, Ms4a14, Ms4a7, Mt1, Mturn, Mvb12b, Mxd1, Mxra7, Myh10, Myh11, Myl9, Mylk, Myo1b, Myo1e, Myom1, Nab2, Nbl1, Ncapg2, Nckap1, Ncoa4, Ndc1, Ndfip1, Ndst1, Nedd4, Negr1, Nek6, Neo1, Neurl1a, Neurl3, Nfe2l2, Nfkbid, Ngfrap1, Nlrp1c-ps, Notch4, Nphp1, Npl, Nr1h3, Nt5c3b, Ntn4, Ntpcr, Nuak1, Nudt9, Odc1, Olfml3, Ophn1, Osbpl1a, Osm, Otub2, P2rx4, P2rx6, P2rx7, P2ry12, P2ry13, P2ry14, P2ry6, P3h2, P3h3, Paip2b, Palb2, Pald1, Palld, Paqr9, Pcx, Pcyt1b, Pdcd1lg2, Pde3b, Pde4b, Pdgfa, Pdgfb, Pdlim4, Pea15a, Pecr, Per1, Pf4, Pfkfb3, Pgf, Phf20, Pianp, Pigp, Pigz, Pilra, Pim3, Pkd1l2, Plcb2, Plcl1, Pld2, Pld3, Plekhg5, Plgrkt, Plk2, Plk3, Plpp3, Plvap, Plxdc2, Plxna3, Plxna4, Plxnb3, Pmaip1, Pmepa1, Pnrc1, Polq, Pon3, Pou6f1, Ppargc1b, Ppp1r12b, Ppp1r13b, Ppp1r15a, Ppp1r16b, Ppp3cc, Prdm1, Prim2, Prkar1b, Prnp, Procr, Prpf40b, Prr5, Prss30, Prune2, Psd3, Ptgs1, Ptgs2, Ptms, Ptp4a1, Ptpdc1, Ptpn23, Ptprv, Ptrf, PVRIG, Pvt1, Pxdc1, Rab13, Rab20, Rab39, Rab3il1, Rab4a, Rad18, Ralgds, Ralgps1, Ralgps2, Rap1gap, Rasal3, Rasgrf2, Rasgrp1, Rbfox2, Rccd1, Rcn3, Rec8, Reps2, Rfx5, Rgl1, Rgs1, Rgs10, Rgs12, Rhbdf1, Rhob, Rhobtb1, Rhoh, Rilpl2, Rnf122, Rnf180, Rogdi, RP23-220F20.2, RP23-312E2.1, RP24-92F8.4, Rpa1, Rpgrip1, Rtn1, Rundc3b, Runx1, Runx2, S1pr1, Saraf, Sash1, Sbf2, Scamp1, Scamp5, Scimp,S cn1b, Scn2b, Sdc4, Selk, Selm, Sema4c, Sema6d,Sepn1, Sepp1, Sept8, Serpina3f, Serpinb8, Serpinb9, Serpine1, Serping1, Sfmbt1, Sh3bp4, Sh3d19, Sh3pxd2a, Sh3pxd2b, Shmt1, Siae, Siglece, Siglecf, Ska2, Skil, Skp2, Slamf6, Slamf9, Slc11a2, Slc12a5, Slc15a3, Slc16a6, Slc19a1, Slc23a2, Slc25a10, Slc25a33, Slc26a11, Slc29a1, Slc2a8, Slc30a4, Slc35b2, Slc37a2, Slc39a8, Slc3a2, Slc44a1, Slc6a8, Slc7a4, Slc7a8, Slc9a3r2, Slc9a9, Slco2b1, Slco3a1, Slit1, Smad7, Smim3, Smo, Smpd3, Smyd4, Snhg15, Snn, Snx24, Snx5, Snx8, Soga1, Sorbs3, Sparc, Specc1l, Spg20, Spint1, Spire1, Spon1, Spred1, Spsb1, Spty2d1, Src,Srxn1, St14,St18, St3gal6, Stab1, Stau2, Stk17b, Stra6l, Stx12, Stxbp1, Sult1a1, Susd1, Syne1, Tacc2, Taf9b, Tagln, Tanc1, Tanc2, Tbc1d10a, Tbc1d16, Tbc1d19, Tbc1d9, Tbrg1, Tbxas1, Tdrkh, Tesk1, Tex9, Tfrc, Tgfbr1, Tgfbr2, Thbs1, Timp3, Tiparp, Tk1, Tle2, Tle6, Tlr1, Tlr12, Tlr3, Tm4sf19, Tmcc3, Tmem106c, Tmem120b, Tmem132a, Tmem201, Tmem206, Tmem231, Tmem26, Tmem37, Tmem82, Tmem86a, Tmem88, Tmem8b, Tmem9, Tmx1, Tnfaip3, Tnfrsf11a, Tnfsf13b, Tnni3, Tns1, Tpbgl, Tpcn2, Tpm2, Trf, Trim37, Trim47, Trim7, Trip13, Trp53bp1, Trp53i11, Trp53inp2, Tsc22d1, Tsen2, Tsku, Tspan3, Tspan33, Tspan4, Tspyl2, Ttc21b, Ttc28, Ttc3, Ttc39b, Ttc8, Ttyh2, Tubb2a, Twsg1, Txlnb, Uap1l1, Ubc, Ube2t, Uevld, Uhrf1bp1, Ulk2, Unc13b, Unc5b, Usp11, Ust, Vav2, Vcam1, Vegfb, Vil1, Vipr1, Vprbp, Vps18, Vwf, Wbp5, Wdr6, Wdr60, Wdr90, Wwp1, Xrcc5, Xylt2, Zbtb10, Zbtb4, Zcwpw1, Zfp334, Zfp36, Zfp438, Zfp523, Zfp667, Zfp691, Zfp90, Zgrf1, Zmynd15, Znrf3, Zranb3, Zscan18*

Table S2. **Gene lists by cluster *(Continued)***

Cluster	Genes
Cluster II	*0610040J01Rik, 1110008F13Rik, 1600014C10Rik, 2310022A10Rik, 2610507I01Rik, 4833407H14Rik, 6430548M08Rik, 9330175E14Rik, A430078G23Rik, A530064D06Rik, AB124611, Abca3, Abca6, Abca7, Abcc4, Abcd2, Abtb1, Acaa2, Acap3, Ace, Acss2, Add3, Adipor2, Adssl1, AI467606, Aim1, Akap13, Akt1, Alas2, Aldh1b1, Aldh2, Amotl2, Ampd3, Ankle2, Anxa7, Ap1m1, Ap1s2, Ap3m1, Apbb1ip, Apobr, Aprt, Arhgap23, Arhgap24, Arhgap26, Arhgap30, Arhgap9, Arhgdib, Arhgef1, Arhgef10l, Arhgef37, Arhgef4, Arid3a, Arl6ip5, Armc3, Arpc5, Arpc5l, Arsg, Asap1, Atg4d, Atg7, Atp1b3, Atp2a3, Atp2b4, Atrnl1, B3galnt2, B3galt4, B430306N03Rik, BC028528, BC051226, Bex6, Bid, Bin2, Bmf, Brpf1, Btla, C1galt1, C1galt1c1, C3, Camkk2, Capn2, Card19, Card9, Casp2, Cast, Cbx8, Ccdc125, Ccdc180, Ccdc88c, Ccnj, Cd177, Cd180, Cd300lg, Cd37, Cd52, Cdc42ep3, Cdc42ep4, Cdc42se1, Cdkn2d, Ceacam1, Ceacam19, Cecr5, Celsr3, Cgnl1, Chfr, Chil3, Chst12, Ckap4, Clec2i, Clmn, Cmas, Cmpk1, Cnr2, Cpt1a, Crybb1, Ctdspl, Ctps2, Ctse, Cyp2ab1, Cyp39a1, Cyp4f16, D1Ertd622e, Daxx, Dbi, Dbnl, Ddit4, Ddx28, Def6, Degs1, Dgka, Dgkg, Diaph1, Dleu2, Dna2, Dnah12, Dnah8, Dock2, Dstn, Dusp3, Dyrk3, E2f2, Ear2, Eif2ak2, Eif4ebp1, Elmo2, Emilin2, Eno1, Eno3, Esyt1, Ethe1, F10, F13a1, F5, Fam129b, Fam160a2, Fam32a, Fam49a, Fam65b, Fam83a, Fam96a, Fas, Fbxl2, Fbxl5, Fbxo4, Fem1a, Fgr, Fis1, Fkbp1b, Flna, Flot2, Flt1, Fmnl1, Fn1, Frat1, Frat2, Fry, Fut7, Fzd4, G6pdx, Gad1-ps, Galns, Gda, Glud1, Gm10384, Gm14326, Gm14548, Gm16194, Gm1673, Gm17354, Gm17767, Gm20559, Gm21975, Gm28875, Gm8369, Gm9733, Gngt2, Gpr141, Gpr15, Gramd3, Grk5, Grk6, Gucd1, Gyg, H13, H2-T24, H3f3a, Hba-a1, Hbb-bs, Hdac10, Helz2, Herc4, Hgsnat, Hhex, Hlcs, Hmha1, Hopx, Hp, Hsd11b1, Hsd17b10, Hsh2d, I830127L07Rik, Ict1, Ifit1bl1, Ifitm3, Ifitm6, Ifngr1, Ikbkb, Ikzf1, Il17ra, Il31ra, Inf2, Ip6k3, Ipmk, Itga1, Itgb2, Jazf1, Kcnab2, Kcnq3, Kctd10, Kctd20, Klc1, Klf10, Klf13, Klhl12, Klhl2, Klhl5, Klrb1f, Kpna2, Krt80, Kxd1, L1cam, Lamtor4, Lbp, Lgals3, Lilra6, Lmf1, Lmo1, Lmo4, Lpcat4, Lrp1, Lrrc20, Lrrc8b, Lrrfip2, Ly6c2, Ly6i, Lyl1, Lyz2, Map2k1, Map4k4, Mars2, Mboat7, Mbp, Mcemp1, Mcf2l, Mcrs1, Me2, Megf9, Met, Mettl7a1, Mfsd14b, Mfsd7a, Mfsd7b, Mgst1, Mid1ip1, Mmp8, Mocs1, Mpnd, Mrpl30, Mrpl33, Ms4a4c, Ms4a8a, Msantd3, Msn, Msrb1, Myo18a, Myo1f, Myo1g, Myo1h, Nadk, Naip2, Ndrg1, Ndufaf7, Nedd9, Nfam1, Nfe2, Nhsl2, Nkiras2, Nsf, Nupr1, Nxpe1-ps, Oas3, Obfc1, Ogt, Orai2, P2ry1, Padi4, Paqr3, Pgam1, Pgd, Pgk1, Pi16, Pik3r6, Pitpna, Pkm, Plcb1, Plcg2, Plec, Plod3, Plp2, Plxnb2, Pnkp, Polr2e, Ppp1r15b, Ppp1r2, Ppp2r5a, Pqlc3, Prkab2, Prkce, Prkch, Prkx, Prr13, Prr5l, Prrc1, Prtn3, Psma7, Psmb3, Psmd8, Pstpip1, Ptgir, Ptpn1, Ptpro, Pyhin1, Rab27a, Rab29, Rab36, Rab3d, Rab44, Rabgap1l, Rac2, Rap1a, Rap2a, Rara, Rarg, Rasa3, Rasgrp2, Rasgrp4, Rassf5, Rbks, Rbm43, Rem1, Rgs14, Rgs19, Rhoq, Rnf144a, Rras, S100a4, S100a6, S1pr4, S1pr5, Samhd1, Sbk1, Sell, Sephs2, Sept9, Serpinb10, Serpinb1a, Sfxn3, Sh2d3c, Sh3bp5, Shfm1, Shisa5, Sigmar1, Sipa1l3, Sirpb1b, Sirpb1c, Slc22a15, Slc22a4, Slc25a37, Slc35c1, Slc44a2, Slc8b1, Slfn1, lfn2, Slfn3, Slfn4, Slfn5, Slfn8, Slk, Slpi, Smim5, Smpdl3a, Snap29, Sntb2, Snx1, Sort1, Sp100, Spata13, Spn, Spryd4, Spsb4, St3gal4, Stat5a, Stat5b, Stk10, Stk24, Stk38, Stom, Stx11, Svil, yne3, Taldo1, Tbc1d2, Tcof1, Tdrd7, Thap11, Ticam2, Tirap, Tm9sf4, Tmc8, Tmem156, Tmem164, Tmem165, Tmem167, Tmem184b, Tmem38b, Tmem43, Tmem71, Tmpo, Tnfrsf21, Tnfrsf8, Tnfsf13, Tnfsfm13, Tpd52, Trem3, Trim16, Trim30b, Tspan14, Tspan17, Tspan5,T spo, Tstd3, Ttc7, Tusc1, Tyk2, Ugt1a7c, Uhrf1bp1l, Unc119, Utp14a, Vamp1, Vcan, Vnn3, Ywhab, Zbtb42, Zfc3h1, Zfp217, Zfp706, Zfp971, Znhit1*
Cluster III	*0610009O20Rik, 2810417H13Rik, Abca1, Alms1, Ankrd26, Arf4, Arhgap22, Arhgap5, Arhgef39, Arhgef40, Arl5b, Asf1b, Atf3, Birc5, Blnk, Brca1, Btbd3, Btg1, Bub1, Casc5, Cass4, Ccdc112, Ccl2, Ccl5, Ccnb1, Ccnb2, Ccnd1, Ccnf, Ccrl2, Cd14, Cd36, Cd69, Cd74, Cdc20, Cdca2, Cdca3, Cdca8, Cdk1, Cenpe, Cenpf, Cep55, Chaf1a, Chd5, Cit, Ckap2l, Ckb, Clec10a, Clec4a2, Clec7a, Coq10b, Csrnp1, Csrp1, Ctnnd2, Cxcr3, Cxcr4, Dbf4, Dlgap5, Dnajb4, Dnajb9, Dnmt3a, Dok2, Dsn1, Dusp10, Dusp2, Dusp5, E230013L22Rik, Ect2, Efhd2, Egr2, Eng, Errfi1, Etf1, Etv3, Exo1, Fanci, Fcrls, Fhl3, Fignl1, Fnbp1l, Fndc7, Foxm1, Gins2, Gm29340, Gm43814, Gpr132, Gpr84, Gpsm1, Grasp, Gstcd, H2-Aa, H2-Ab1, H2-DMa, H2-DMb1, H2-DMb2, H2-Eb1, H2-Ob, H3f3b, Hbegf, Hdac9, Hepacam2, Hmmr, Ier2, Ier5, Ifrd1, Igsf9, Il1a, Il1b, Il1rl1, Il1rn, Il6, Il7r, Ildr1, Itga6, Itgae, Itpkc, Kcnd1, Kdm6b, Kif11, Kif14, Kif15, Kif18b, Kif20a, Kif20b, Kif22, Kif2c, Kif4, Kifc1, Kit, Klf6, Klri1, Knstrn, Kpna3, Lig1, Lmnb2, Lrrc3, Ltbp3, Maff, Map3k8, March3, Marcksl1, Mcm2, Mcm3, Mcm5, Mcm6, Mcm7, Melk, Mki67, Mmp12, Mns1, Myc, Mycl, Ncapd2, Ncapg, Ncaph,Ndc80, Nectin2, Nek2, Nfatc2, Nfil3, Nfkbia, Nfkbie, Nfkbiz, Nlrp3, Nr4a1, Nr4a2, Nrip1, Nuf2, Nusap1, Nxpe5, Oat, Ocrl, P2ry10, Pak1, Paqr4, Phlda1, Phldb1, Pim1, Pkmyt1, Plau, Plek, Plk1, Plk4, Pltp, Pola1, Pold1, Pole, Ppfibp1, Prc1, Prim1, Ptger3, Ptpn7, Rab43, Rab6b, Rab7b, Rabgef1, Rad51, Rad51ap1, Rad54l, Rai14, Rapsn, Rbpj, Rel, Rnf141, Rnf150, Rnf19b, Rrad, Rrm1, Rrm2, Runx3, Serpinf1, Sgol2a, Shcbp1, Shmt2, Slc20a1, Slc43a3, Slc4a7, Smc2, Socs3, Sox12, Spc24, Spc25, Spdl1, Sqstm1, Stil, Stmn1, Syce2, Tacc3, Tagap, Tgif1, Tgif2, Tgoln1, Thap6, Ticrr, Tie1, Tlcd2, Tmem119, Tmem176a, Tmem176b, Tnf, Tnfsf8, Tob2, Top2a, Tpx2, Trib1, Troap, Tspan32, Tuba1c, Tubb5, Tubb6, Tyms, Uhrf1, Ung, Vegfa, Wnk1, Zc3h12a, Zfand5, Zfp36l1, Zfp395, Zfp703, Zwilch*
Cluster IV	*4930523C07Rik, A630033H20Rik, AA414768, Abhd5, Acot11, Acot7, Adam15, Adam8, Adgre5, Adora2a, Adss, Agpat2, Agpat4, Ahnak, AI839979, Alox5ap, Amd1, Ankrd28, Anxa1, Anxa2, Api5, Arhgap15, Arpc2, Atp10a, Atp11b, Atp5c1, B4galt5, Bbc3, BC005537, BC147527, Bcl11a, Bcl6, Bcr, Bri3bp, Bst1, Btf3, Bysl, Capg, Ccdc109b, Ccl6,Ccl9, Ccnd3, Cd300lb, Cd47, Cers6, Clec12a, Clec4a3, Clint1, Cmip, Cmpk2, Cnn2, Coq2, Coro1a, Crip1,C sgalnact2, Dennd4a, Diaph2, Dock8, E230016K23Rik, E330009J07Rik, Eif1ax, Eif3f, Eif3h, Eif3k, Eif3m, Emb, Eml2, Emp3, Eps8, Fam107b, Fam111a, Fam117b, Fam129a, Fam69a, Fam78b, Fam83f, Fam84b, Fcho1, Foxk1, Foxn2, Furin, Fxyd5, G430095P16Rik, Galnt9, Gapt, Gch1, Glipr2, Gltscr2, Gm11772, Gm13373, Gm15987, Gm1966, Gm43197, Gm8995, Gng10, Got2, Gpr35, Gpx1, Gria3, Gspt2, Gtf2h3, Gvin1, Gypc, Haao, Hpse, Icam2, Ifi27l2a, Il6ra, Impa2, Iqgap1, Iqgap2, Irak3, Itga4, Itga5, Itgb7, Itpr1, Jarid2, Kbtbd11, Kctd1, Klf8, Kmt5a, Lbr, Ldhb, Ldlrad3, Limd1, Lpl, Lsp1, Lsr, Ltb4r1, Lyst, Map3k6, Map4k1, Map7, Mbnl3, Mboat1, Mfsd6, Mllt4, Mmp28, Mob3a, Mob3b, Ms4a4b, Mthfd1l, Myadm, N4bp1, Naaa, Naca, Napsa, Nat8l, Nbeal2, Ncor2, Nfkb1, Nin, Nrm, Nsa2, Padi2, Pdha1, Pdlim1, Pfkp, id1, Piezo1, Pkp3, Plac8, Plekhf2, Plekhg1, Plpp2, Polr1a, Prdx6, Prkag2, Ptgr1, Pygl, Rab31, Ramp1, Rap1gap2, Rassf3, Rassf4, Rbpms, Rell1, Rfx2, Rplp0, Rps15a, Rps27a, Rpsa, Ryr1, S100a10, S100a11, Samd9l, Samsn1, Sdad1, Selplg, Sept6, Sgms2, Sigirr, Sirpb1a, Siva1, Slc16a10, Slc16a3, Slc25a24, Slc2a6, Slc35e4, Slc52a3, Smpdl3b, Snrk, Sntb1, St3gal1, Steap3, Stk26, Stx3, Stxbp6, Sun1, Susd3, Svip, Tarm1, Thbd, Tiam1, Tiam2, Tkt, Tle3, Tmcc1, Tmem154, Tmsb10, Tnfsf13os, Tnfsf14, Tppp3, Traf3ip2, Trem2, Treml2, Trps1, Tuba1a, Unc119b, Upb1, Vcl, Vim, Vps13c, Wdr3, Xylt1, Ybx3, Zak, Zyx*

Table S2. **Gene lists by cluster *(Continued)***

Cluster	Genes
Cluster V	*1700017B05Rik, 2610203C22Rik, Abca5, Abcb1a, Acpp, Acy3, Ada, Adam33, Adamdec1, Adcy4, Adcy6, Adgb, Adrb1, Aga, Agpat3, Akr1b10, Aldh1l1, Aldh7a1, Als2cl, Angptl4, Ankrd24, Aplp1, Appl2, Arhgap10, Arhgef5, Arl4d, Armc8, Asap3, Aspa, AW011738, B3galt5, B4galnt4, B4galt4, Bach2os, Bcl7a, Bco2, Blvrb, Btnl7-ps, Cabp4, Camk2n1, Catip, Ccdc88b, Ccl24, Ccr1l1, Cd163, Cd200r1, Cd4, Chst14, Clip2, Clstn1, Cntrob, Col11a2, Coro2b, Cplx2, Cracr2b, Cryz, Csf1r, Csf2rb2, Cwc25, Cysltr2, Dcbld2, Dchs1, Dcun1d4, Dhcr7, Dkk3, Dlc1, Dnajc16, Dock9, Dpy19l3, Dsel, Dtnb, Dtx3, E230029C05Rik, Ecm1, Efemp2, Ehf, Eif2b2, Engase, Enpp2, Epb41l3, Epor, Esam, Eya4, Fam135a, Fam13a, Fam189a2, Fap, Fer, Firre, Fkbp14, Folr2, Fpr1, Fsd2, Galm, Ganc, Gas1, Gatsl3, Ggta1, Gkap1, Gm26947, Gm28884, Gng7, Gpm6b, Gpnmb, Gpr157, Gstm2, H2-M2, Havcr2, Hist1h1c, Hist1h2bc, Hjurp, Hoxb3, Hoxb5, Hsd17b7, Hunk, Idua, Igf2bp3, Ikzf2, Kcnj16, Kcp, Kctd6, Kif5c, Klhl13, Klra17, Kmo, Krba1, Lbh, Leprot, Lrp4, Lrrc4c, Man1c1, Marveld2, Mcfd2, Mdfi, Med12l, Mfsd12, Mok, Mpp6, Mpzl2, Mroh2a, Mxra8, Mycbpap, Myo7a, Nceh1, Nckap5l, Neil2, Nfxl1, Nhlrc3, Ninl, Nisch, Nphp3, Npr1, Nucb2, Nudt16, Ocstamp, Odf2l, Oplah, Optn, Osbpl10, Osgin1, P2rx1, Paox, Pccb, Pcdhgb2, Pde1b, Pdgfc, Pecam1, Peli3, Phactr1, Phkg1, Pla2g15, Pla2g2d, Pla2g7, Plaur, Plcd1, Plekha8, Plekhm2, Plpp5, Pmvk, Ppfibp2, Prkca, Pter, Ptpn13, Pyroxd2, Rab11fip3, Rab11fip5, Rab34, Rcbtb2, Rell2, Renbp, Rgmb, Rhobtb3, Rhoc, Rnasel, Rnf43, RP24-185N3.3, Rsad1, Scarf2, Sema4b, Sept4, Sgk1, Sipa1l1, Slc12a2, Slc28a2, Slc38a6, Slc40a1, Slc44a5, Slc46a1, Slc4a8, Slc5a6, Slit3, Smad6, Smagp, Snx18, Spats2, Spry1, Srcin1, St5, Stard13, Svbp, Syt3, Tbc1d12, Tfpi, Thnsl2, Thrb, Tmeff1, Tmem144, Tmem150b, Tmem2, Tmem87a, Tmem87b, Tpp1, Trim6, Trpm2, Trpv4, Tspan13, Tspan8, Tssc4, Tuft1, Ubald1, Wnt4, Wtip, Zcchc14, Zdhhc1, Zdhhc14, Zfp105, Zfp579, Zfp622, Zfp773, Zfp820, Zkscan7, Zscan20*
Cluster VI	*2410016O06Rik, 2500002B13Rik, 9330151L19Rik, A130014A01Rik, AI480526, Aldh3b1, Alkbh4, Ap5b1, Arhgef18, Atp1a3, Atp6v1b2, Bmx, Bre, Ccdc71l, Cd244, Clec4d, Csf3r, D230025D16Rik, Dclre1b, Dusp7, Gcnt2, Gm15922, Gpld1, Gramd4, Gsr, Gss, Gstm1, Idh1, Igsf6, Itgal, Itgb3, Klhl42, Mdm1, Mirt1, Mlst8, Mocos, Mpp1, Mst1r, Mtus1, Nlrx1, Nod1, Nxpe4, Pira2, Ppp2cb, Rhou, Rspo1, Sash3, Scarb1, Scnn1a, Socs6, Sqrdl, Stk16, Tmc6, Tnfaip8l2, Tnfrsf1b, Traf3ip3, Tuba4a, Xdh, Zfp760, Zfp87, Zfyve9*
Cluster VII	*1700025G04Rik, Adora2b, Akt3, Alcam, Aplnr, Arsb, Ass1, Atp8b4, Ccr2, Clec4e, Coro2a, Eif3e, Esr1, Flt3, Foxred2, Glipr1, Kctd17, Lgals1, Lrrk2, Lyz1, Mthfd2, Nectin1, Nfix, Nup210, Olfm1, Olr1, Parvg, Prkd3, Psat1, Qpct, Rab11fip1, Rgs18, Rnf217, Satb1, Sema4a, Skint3, St3gal5, Synj2, Tnip3, Tpm4, Ttc39c, Uck2, Zbtb18*
Cluster VIII	*Clec4b1, Id3, Retnla, Tnfsf9*
Cluster IV	*AI504432, Emp1, Rgag4, Slamf7, Spp1, Stat4, Traf1*
Cluster X	*Cyfip2, Pstpip2, Rgs3, Wfdc17*
Cluster XI	*Ddhd1, Il12rb2*
Cluster XII	*2900052N01Rik, Adam28, Adcy3, Adgra3, Angptl7, C2, Casc4, Ccsap, Cd209f, Cd209g, Cdc42bpa, Edil3, Fcna, Fkbp9, Fndc5, Fstl4, Fzd8, Gpr137b, Gpx3, Lgr4, P2ry2, Pkp4, Ppp1r9a, Ptpn14, Rims3, Rusc2, Serpinb6a, Slc1a3, Slc22a17, Slco4a1, Smim1, Spats2l, Speg, Spock1, St6galnac3, Tcaf1, Timd4, Uaca, Xlr, Zfp641*
